# Multilayer stabilization for fabricating high-loading single-atom catalysts

**DOI:** 10.1038/s41467-020-19599-8

**Published:** 2020-11-18

**Authors:** Yazhou Zhou, Xiafang Tao, Guangbo Chen, Ruihu Lu, Ding Wang, Ming-Xi Chen, Enquan Jin, Juan Yang, Hai-Wei Liang, Yan Zhao, Xinliang Feng, Akimitsu Narita, Klaus Müllen

**Affiliations:** 1 Max Planck Institute for Polymer Research, 55128 Mainz, Germany; 2 School of Materials Science and Engineering, Jiangsu University, Zhenjiang, Jiangsu 212013 China; 3 Center for Advancing Electronics Dresden (Cfaed) and Faculty of Chemistry and Food Chemistry, Technische Universität Dresden, 01062 Dresden, Germany; 4 State Key Laboratory of Silicate Materials for Architectures, International School of Materials Science and Engineering, Wuhan University of Technology, Wuhan, Hubei 430070 China; 5 Hefei National Laboratory for Physical Sciences at the Microscale, Department of Chemistry, University of Science and Technology of China, Hefei, 230026 China; 6 Organic and Carbon Nanomaterials Unit, Okinawa Institute of Science and Technology Graduate University, Okinawa, 904-0495 Japan

**Keywords:** Electrocatalysis, Synthesis and processing

## Abstract

Metal single-atom catalysts (M-SACs) have emerged as an attractive concept for promoting heterogeneous reactions, but the synthesis of high-loading M-SACs remains a challenge. Here, we report a multilayer stabilization strategy for constructing M-SACs in nitrogen-, sulfur- and fluorine-co-doped graphitized carbons (M = Fe, Co, Ru, Ir and Pt). Metal precursors are embedded into perfluorotetradecanoic acid multilayers and are further coated with polypyrrole prior to pyrolysis. Aggregation of the metals is thus efficiently inhibited to achieve M-SACs with a high metal loading (~16 wt%). Fe-SAC serves as an efficient oxygen reduction catalyst with half-wave potentials of 0.91 and 0.82 V (versus reversible hydrogen electrode) in alkaline and acid solutions, respectively. Moreover, as an air electrode in zinc–air batteries, Fe-SAC demonstrates a large peak power density of 247.7 mW cm^−2^ and superior long-term stability_._ Our versatile method paves an effective way to develop high-loading M-SACs for various applications.

## Abstract (web summary)

Metal single-atom catalysts offer great potential in bridging the gap between heterogeneous and homogeneous catalysis. Here the authors demonstrate a multilayer stabilization strategy for fabricating high-loading single-atom catalysts including non-precious and noble metals.

## Introduction

Heterogeneous metal catalysts play a vital role in industrial chemical synthesis, sustainable energy conversion devices and advanced biotechnologies^1–4^. A key concept for catalyst design is the atomic dispersion of metals on solid supports, leading to so-called single-atom catalysts (SACs)^5,6^. Single-metal atoms hold promise for unique activity, selectivity, and atom utilization efficiency^7,8^. The supports can also change the electronic structures of the single atoms. SACs can thus provide versatile pathways for efficient mass and charge transport, and bridge the gap between homogeneous and heterogeneous catalysis^9,10^.

There is ample literature evidence that the single-atom character^11,12^, coordination environment^13,14^, metal-support interaction^15–17^, and metal loading are important factors that determine the catalytic performance of SACs^18^. Increasing the metal loading while maintaining the atomic dispersion should be a promising strategy to further enhance catalytic performance^19^. However, the surface free energy of metals increases significantly with decreasing particle size, leading to the successive formation of clusters and nanoparticles with a lower activity^5^. Various concepts have been proposed to fabricate SACs through the stabilization of metal atoms on supports^20,21^, such as defect engineering of metal hydroxide and oxide supports^22^, spatial confinement in zeolites^23,24^, fabrication of metal-organic frameworks (MOFs) or covalent-organic frameworks^25–27^, and utilization of strong interactions between metal species and coordinating heteroatoms (e.g., nitrogen (N), oxygen (O), and sulfur (S))^28–30^. For example, Zhang et al.^20^ demonstrated that defects in nickel hydroxide (Ni(OH)_*x*_) could serve as ‘traps’ to stabilize metal species, achieving a Pt loading much higher than that attained by using defect-free Ni(OH)_*x*_. Li et al.^31^ employed zeolitic imidazolate frameworks (ZIFs), a class of MOFs as anchoring sites, to stabilize single manganese (Mn) atoms with a Mn content of 3.03 wt%. However, these approaches still do not rigorously exclude metal aggregation. This drawback is especially severe when using high amounts of metal precursors and high-temperature pyrolysis, because acidic etching processes to remove the non-single metallic species are required^32^. These procedures not only make the control of metal loading challenging but also randomly create single atomic active sites on the supports, resulting in low reproducibility^33^. Moreover, most of the reported SACs have very low metal loading, so robust approaches for fabricating SACs with high metal loading (>2 wt%) have remained elusive^19^.

Here we report a uniform protocol for multilayer stabilization to produce high-loading M-SACs supported by N, S, and fluorine (F)-co-doped porous graphitized carbon (M-SA-NSFC). Our concept comprises confinement of organometallic precursors (OMs) inside perfluorotetradecanoic acid (PFTA) bilayers that are further coated with polypyrrole (Ppy) layers prior to a final pyrolysis process. The confinement by PFTA and Ppy multilayers can efficiently prevent OMs from migrating during the pyrolysis process, resulting in the coordination of isolated metal atoms with N atoms in porous graphitized carbon. This method enables the synthesis of various M-SACs with a high loading of up to ~16 wt%, including both non-precious and noble metals of iron (Fe), cobalt (Co), ruthenium (Ru), iridium (Ir), and platinum (Pt). Furthermore, the electrocatalytic oxygen reduction reaction (ORR), a crucial process in next-generation energy storage and conversion systems, is employed to investigate the potential application of Fe-SA-NSFC. As the result, the Fe-SA-NSFC achieves high half-wave potentials (*E*_1/2_) of 0.91 and 0.82 V (vs. reversible hydrogen electrode (RHE)) towards the ORR in alkaline and acid electrolyte solutions, respectively, which outperforms those values for the most state-of-the-art noble metal-free electrocatalysts. Furthermore, as an air electrode, the Fe-SA-NSFC shows a good performance in a zinc (Zn)-air battery, including a large peak power density of 247.7 mW cm^−2^ and a long-term stability over a period of 240 h. Besides, the Co-SA-NSFC shows excellent activity toward hydrogen evolution reaction (HER) in acidic electrolyte solution, which is the most sustainable pathway for hydrogen production.

## Results

### Catalyst synthesis

**Fig. 1:**
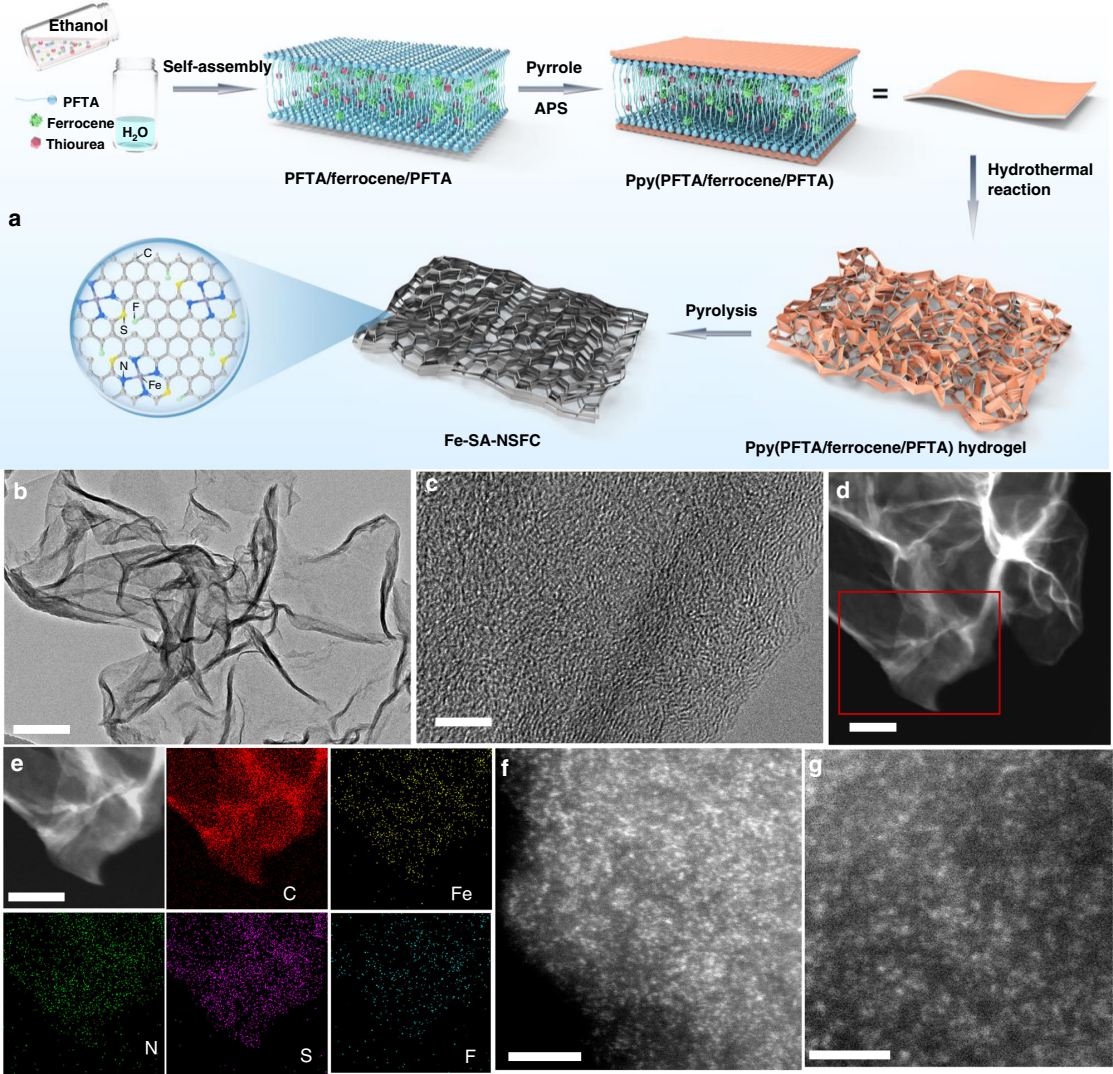
Synthesis and structural characterization results. **a** Illustration of the preparation process for the Fe-SA-NSFC and **b** TEM, **c** HRTEM, and **d** HAADF-STEM images of the Fe-SA-NSFC. **e** Enlarged HAADF-STEM image and corresponding element maps for C, Fe, N, S, and F. **f** AC-HAADF-STEM image and **g** high-magnification AC-HAADF-STEM image of the Fe-SA-NSFC. Scale bars: **b** 200 nm, **c** 5 nm, **d** 200 nm, **e** 100 nm, **f** 5 nm, and **g** 2 nm.

Fe-SA-NSFC was synthesized as an example to describe the multilayer stabilization used to fabricate a M-SAC, as illustrated in Fig. 1a (see details in ‘Methods’ and Supplementary Fig. 1). In a typical experiment, ferrocene (0.2 mmol), thiourea (0.7 mmol), and PFTA (0.07 mmol) were dissolved in 2 mL of ethanol. Then, water (10 ml) was added to induce the self-assembly of PFTA towards lamella-type bilayers with hydrophilic surfaces and hydrophobic interlayers^34^. The PFTA bilayers had a sheet-like morphology with sizes up to 4 μm^34,35^. The ferrocene was simultaneously confined into the interlayers of the PFTA to achieve PFTA/ferrocene/PFTA layers due to its high hydrophobicity. To further stabilize the ferrocene in the PFTA/ferrocene/PFTA bilayers, the PFTA surfaces were coated with Ppy layers by adding pyrrole (124 μL), which was adsorbed due to hydrogen bonding^35^. Oxidative polymerization of the pyrrole with ammonium persulfate (APS) gave rise to the formation of sheet-like Ppy(PFTA/ferrocenes/PFTA) (Supplementary Fig. 2). Subsequently, the Ppy(PFTA/ferrocene/PFTA) was hydrothermally treated at 150 °C for 8 h, establishing a free-standing hydrogel through cross-linking of adjacent Ppy layers (Supplementary Fig. 1d)^34^. The obtained hydrogel was freeze-dried and then pyrolysed at 1000 °C for 1 h under an Ar atmosphere for carbonization. After cooling to room temperature, Fe-SA-NSFC was produced as a black powder without any further treatments (Supplementary Fig. 1e).

### Catalyst characterization

Visualization by bright-field and medium-angle annular dark-field scanning transmission electron microscopy (STEM) and scanning electron microscopy (SEM) indicated the sheet-like morphology of the Fe-SA-NSFC, which was inherited from the PFTA bilayers (Fig. 1b and Supplementary Fig. 2). The powder X-ray diffraction (XRD) pattern of the Fe-SA-NSFC showed only one peak at ~25° corresponding to the (002) plane of graphite (Supplementary Fig. 3a) and its Raman spectrum exhibited D and G bands at 1342 and 1585 cm^−1^ (Supplementary Fig. 4), respectively, indicating the formation of graphitized carbon^27^. High-resolution transmission electron microscopy (HRTEM) images together with XRD verified the absence of crystalline Fe-based nanoparticles or clusters in the Fe-SA-NSFC (Fig. 1c and Supplementary Fig. 3a). The corresponding element mapping images revealed a homogeneous distribution of C, N, S, F, and Fe in the Fe-SA-NSFC (Fig. 1d, e). Furthermore, aberration-corrected high-angle annular dark-field STEM (AC-HAADF-STEM) images showed that numerous separated Fe atoms with an average diameter of ~0.17 nm were uniformly distributed over all of the graphitized carbon supports (Fig. 1f, g and Supplementary Fig. 5). No larger particles or crystalline Fe phases were observed, validating that the identified Fe atoms were present as single atoms. The Fe loading in the Fe-SA-NSFC reached 15.3 wt% when 0.2 mmol of ferrocene was used, as determined by inductively coupled plasma mass spectrometry (ICP-MS) analysis. This loading is higher than that of the most of reported Fe-SACs (Supplementary Table 1)^3,8,19^. A continued increase in the amount of ferrocene (e.g., 0.25 mmol) resulted in the generation of Fe-containing nanoparticles (NPs) and clusters loaded on the N, S, F-co-doped graphitized carbon (Fe-NSFC), as evidenced by XRD and TEM analyses (Supplementary Figs. 3a and 6). In addition, control experiments (i) in the absence of PFTA and (ii) by using water-soluble Fe precursors instead of ferrocene (e.g., ferrous sulfate heptahydrate, FeSO_4_·7H_2_O) failed to achieve the atomic dispersion of Fe at high loadings pyrolysis (Supplementary Fig. 7). This indicated, again, the excellent ability of the current multilayer confinement to suppress the formation of Fe-containing NPs or clusters during high-temperature pyrolysis.

**Fig. 2:**
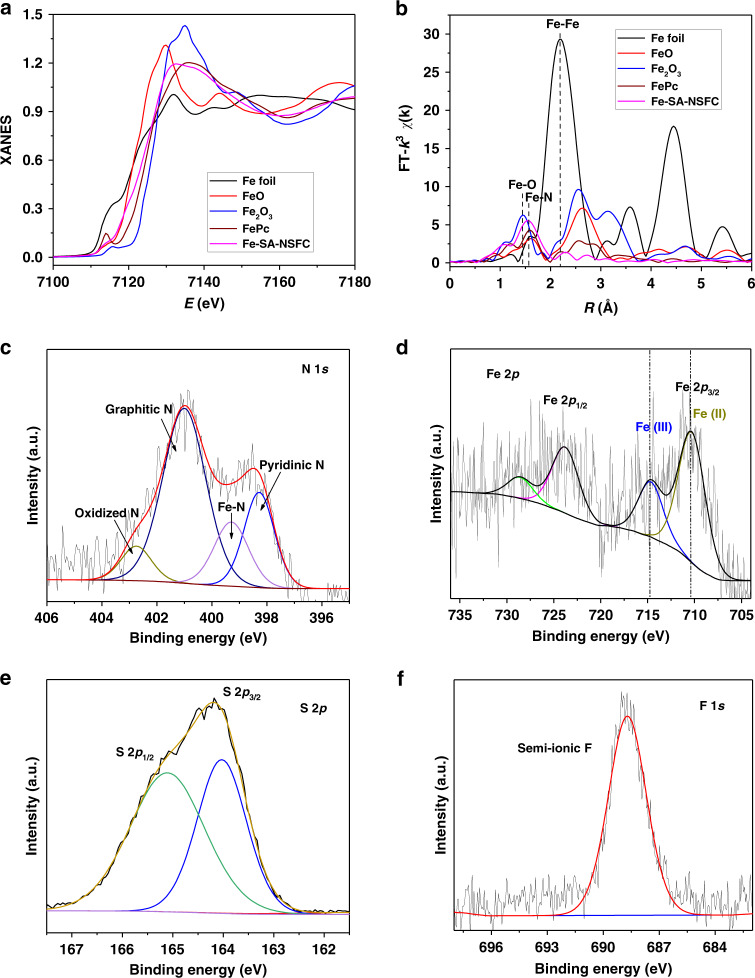
Atomic structure analysis. **a** Fe K-edge XANES spectra and **b** FT of *k*^3^-weighted *χ*(*k*)-function of EXAFS spectra of Fe-SA-NSFC, where an Fe foil, FeO, Fe_2_O_3_, and FePc were used as reference materials. High-resolution X-ray photoelectron spectroscopy (XPS) spectra of **c** N 1*s*, **d** Fe 2*p***, e** S 2*p*, and **f** F 1*s*.

The synchrotron radiation-based X-ray absorption fine spectroscopy (XAFS) results revealed the atomic structure and coordination state of the Fe species. The X-ray absorption near-edge structure (XANES) spectrum of the Fe-SA-NSFC showed that the Fe *K*-pre-edge was close to that for Fe(II) in FeO but far from that for Fe(III) in Fe_2_O_3_, indicating that the oxidation state of the Fe in the Fe-SA-NSFC was +2 (Fig. 2a). The Fourier transform of the *k*^3^-weighted extended X-ray absorption fine structure (EXAFS) spectrum of the Fe-SA-NSFC displayed a clear peak at ca. 1.57 Å (Fig. 2b), which was ascribed to the Fe-N first coordination shell^36^. Compared with the peaks from Fe foil, FeO, Fe_2_O_3_, and FeS_2_ as reference materials, no Fe-Fe (ca. 2.20 Å), Fe-O (ca. 1.45 Å), and Fe-S (ca. 1.80 Å) coordination peaks were detected, in accordance with the atomic dispersion of the Fe^37^. Based on R-space fitting of the EXAFS data, the coordination number of the Fe-N first shell was given by CN_Fe-N_ = 4.4 ± 0.5 (Supplementary Fig. 8 and Supplementary Table 2), suggesting that the dominant Fe-N structure was FeN_4_.

X-ray photoelectron spectroscopy (XPS) analysis revealed the binding states of different elements in the Fe-SA-NSFC, as summarized in Supplementary Tables 3–5. The contents of N, S, and F were 17.2%, 2.5%, and 2.2 at%, respectively (Supplementary Table 3), indicating high doping densities of these heteroatoms. The high-resolution C 1*s* spectrum from the Fe-SA-NSFC showed four peaks at 284.7, 285.8, 288.6, and 291.0 eV, which could be assigned the C=C, C-N (C−S), O=C−O, and C-F bonds, respectively (Supplementary Fig. 9). The N 1*s* spectrum revealed the presence of four types of N species, namely, pyridinic N (398.6 eV), Fe-N (399.5 eV), graphitic N (401.2 eV), and oxidized N (402.5 eV) (Fig. 2c)^18^. The high content of FeN_4_ (15.0%) was attributed to the pronounced N-coordination sites that anchored Fe and enabled the formation of FeN_4_ moieties with a high density. The high-resolution Fe 2*p* XPS spectrum again confirmed the absence of metallic Fe in the Fe-SA-NSFC, and that Fe(II) was the dominant Fe (Fig. 2d)^12^. The S 2*p* and F 1*s* XPS spectra revealed the existence of C-S-C covalent bands and semi-ionic C-F bonds in the Fe-SA-NSFC (Fig. 2e, f)^38,39^.

N_2_ adsorption–desorption analysis was applied to quantify the porous properties of the Fe-SA-NSFC, in which an increased adsorption volume at a low pressure (*P*/*P*_0_ < 0.03) and a distinct hysteresis loop at a high pressure of *P*/*P*_0_ > 0.4 indicated the coexistence of micropores, mesopores, and macropores (Supplementary Fig 10a). The Brunauer–Emmett–Teller specific surface area and total pore volume of the Fe-SA-NSFC were ~900 m^2^ g^−1^ and ~1.2 cm^3^ g^−1^, respectively, which were much larger than those of the Fe-SA-NSFC before the pyrolysis process (~450 m^2^ g^−1^ and 0.4 cm^3^ g^−1^, respectively). A considerable increase in apparent micropores resulted from the carbonization of ferrocene and PFTA at high temperatures. This may be ascribed to the improved porosity of the Fe-SA-NSF, which is consistent with the pore size distribution analysed by a nonlocal density functional theory (DFT) method (Supplementary Fig. 10b).

### Fabrication of M-SA-NSFCs with other metals

**Fig. 3:**
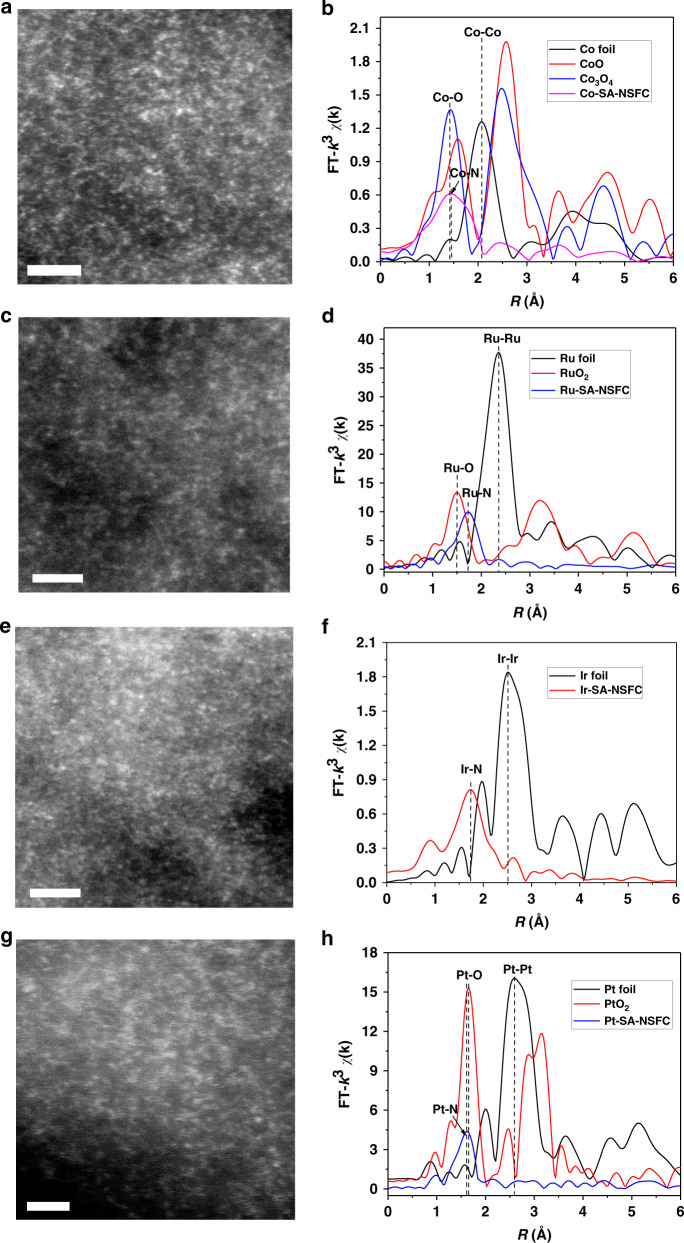
Characterization of M-SA-NSFCs with other metals. **a**, **c**, **e**, **g** AC-HAADF-STEM images and **b**, **d**, **f**, **h** EXAFS experimental data: **a**, **b** Co-SA-NSFC; **c**, **d** Ru-SA-NSFC; **e**, **f** Ir-SA-NSFC; and **g**, **h** Pt-SA-NSFC. Scale bar: 2 nm.

The broad scope of this multilayer stabilization strategy was next examined for the preparation of a series of M-SA-NSFCs with different metals, including Co, Ru, Ir, and Pt, by using the corresponding OMs with an optimal dosage. For noble metals, thermal reduction was performed under a H_2_/Ar atmosphere at 250 °C for 2 h owing to the high reduction ability of H_2_^29^. Similar to the characterization of the Fe-SA-NSFC, the XRD, TEM, HRTEM, elemental mapping, and XPS methods were employed to investigate the crystalline structure, morphology, and element distribution of the obtained M-SA-NSFCs (Supplementary Fig. 3c, d and Supplementary Figs. 11–13). The XPS results revealed the heteroatom-doping structure in noble metal containing catalysts particularly O, N, and S. This structure was different from Fe-SA-NSFC due to the lower annealing temperature (Supplementary Fig. 13). The AC-HAADF-STEM images revealed the atomic dispersion of the metal atoms on the graphitized carbon for the M-SA-NSFCs (M = Co, Ru, Ir, and Pt) (Fig. 3). The EXAFS analyses indicated no metal–metal coordination (Fig. 3). Moreover, the EXAFS results confirmed that all metal atoms were coordinated with N (Supplementary Fig. 14). The first-shell coordination numbers of M-N were 4.8 ± 1.6 for Co, 5.0 ± 0.6 for Ru, 4.0 ± 0.8 for Ir, and 4.0 ± 0.6 for Pt (Supplementary Table 2), corresponding to CoN_5_, RuN_5_, IrN_4_, and PtN_4_, respectively^29,40^. The metal loading of the M-SA-NSFCs could, again, be controlled by adjusting the relative amounts of metal precursors, yielding M-SA-NSFCs with maximum metal loadings of 12.2, 10.9, 15.7, and 15.6 wt% for Co, Ru, Ir, and Pt, respectively. Again, these values are much higher than those reported in most of the previous publications (Supplementary Table 1)^3,10,29,40^.

### Electrocatalytic ORR performance of Fe-SA-NSFC

**Fig. 4:**
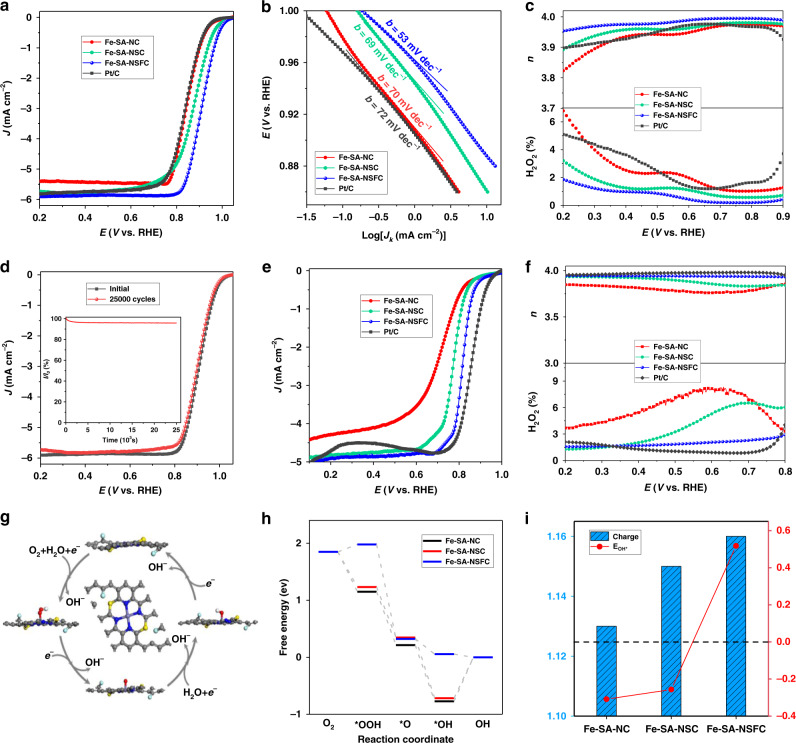
Electrocatalytic ORR performance. **a** ORR polarization curves, **b** Tafel plots, and **c** electron-transfer number (*n*) (top) and H_2_O_2_ yield (bottom) for the Fe-SA-NC, Fe-SA-NSC, Fe-SA-NSFC, and Pt/C in 0.1 M KOH solution. **d** ORR polarization curves of the Fe-SA-NSFC before and after 25000 CV cycles; the inset of **d** shows the *i*–*t* curve at 0.9 V and 1600 r.p.m. for 25,000 s. **e** ORR polarization curves and **f** electron-transfer number (*n*) (top) and H_2_O_2_ yield (bottom) for the Fe-SA-NC, Fe-SA-NSC, Fe-SA-NSFC, and Pt/C in 0.1 M HClO_4_ solution with rotation speed of 900 r.p.m. **g** Proposed 4*e*^−^ reduction mechanism in alkaline solution for the ORR on Fe-SA-NSFC; the inset shows the structure of the Fe-SA-NSFC. **h** Free energy diagram for the Fe-SA-NC, Fe-SA-NSC, and Fe-SA-NSFC (*U* = 0 V vs. RHE, pH 13). **i** OH* binding energies and Mulliken charges of the FeN_4_ active centres in the Fe-SA-NC, Fe-SA-NSC, and Fe-SA-NSFC.

The electrochemical performance of Fe-SA-NSFC for the ORR and the effect of the S and F dopants were studied using rotating disk electrode (RDE) and rotating ring-disk electrode (RRDE) measurements. The prepared SA-Fe supported by N-doped and N,S-doped graphitized carbons (Fe-SA-NC and Fe-SA-NSC, respectively), as well as that for the commercial Pt/C were evaluated for comparison (Supplementary Figs. 15 and 16). Cyclic voltammetry (CV) curves of all the Fe-SACs displayed a clear cathodic ORR peak in an O_2_-saturated 0.1 M KOH electrolyte solution (Supplementary Fig. 17a). The onset potential was defined as the potential required for generating an ORR current density of 0.1 mA cm^−2^ in linear sweep voltammetry (LSV) curves^41^. The Fe-SA-NSFC demonstrated the highest ORR activity herein in terms of the most positive onset potential of 1.01 V vs. RHE (Fig. 4a). The *E*_1/2_ was up to 0.91 V, which surpassed the values for the Fe-SA-NC (0.86 V), Fe-SA-NSC (0.88 V), and Pt/C (0.85 V) (Supplementary Table 6). The Fe-SA-NSFC also presented a comparable ORR activity with most of the reported state-of-the-art Pt-free catalysts, such as the atomic Fe–N_*x*_ moieties anchored on carbon support (Fe–NC SACs) (0.90 V)^36^, Fe‐isolated single atoms on S- and N‐co-doped carbon (Fe-ISA/SNC) (0.896 V)^37^, and N, phosphorus (P), and S co-doped hollow carbon polyhedron (Fe-SAs/NPS-HC) (0.912 V)^42^ (Supplementary Table 7). Moreover, the Fe-SA-NSFC exhibited the highest kinetic current density (*J*_k_) at 0.85 V herein of 61.5 mA cm^−2^ compared to that for the Fe-SA-NC (5.3 mA cm^−2^), Fe-SA-NSC (13.6 mA cm^−2^), and Pt/C catalyst (5.2 mA cm^−2^). The Tafel slope of the Fe-SA-NSFC was measured to be 53 mV dec^−1^, which was lower than the values of 70 mV dec^−1^ for the Fe-SA-NC, 69 mV dec^−^^1^ for the Fe-SA-NSC, and 72 mV dec^−1^ for the Pt/C (Fig. 4b). Accordingly, S and F co-doping greatly accelerated the ORR kinetics for the FeN_4_ active sites in alkaline solution.

To further probe the ORR kinetics of the Fe-SA-NSFC, RDE measurements at different speeds of rotation were performed, and the related Koutecký–Levich (K-L) plots were acquired. Linear and almost parallel K-L plots were achieved at different applied potentials (Supplementary Fig. 17b, c), reflecting first-order reaction kinetics towards the concentration of dissolved oxygen and a similar electron-transfer number (*n*). The electron-transfer number was calculated to be 3.98 at 0.4 V, pointing towards a four-electron (4*e*^−^) oxygen reduction process^27^. An RRDE technique was employed to monitor the generation of H_2_O_2_ during the ORR process with the ring potential of 1.45 V vs. RHE. The H_2_O_2_ yield from the Fe-SA-NSFC was below 2% in the potential range from 0.40 to 0.90 V, implying an excellent selectivity of the oxygen reduction towards H_2_O formation (Fig. 4c).

To evaluate the durability of the Fe-SA-NSFC, accelerated durability tests (ADTs) and *i*–*t* chronoamperometry techniques were used. After 25,000 potential cycles, the *E*_1/2_ of the Fe-SA-NSFC decreased by only 2 mV (Fig. 4d), which was much smaller than that of Pt/C (50 mV) (Supplementary Fig. 17d). A durability test at a constant potential of 0.9 V for 25,000 s at a rotation speed of 1600 r.p.m. showed that only 4.4% of the current density was lost for the Fe-SA-NSFC (Fig. 4d, inset), again confirming the good ORR stability of the Fe-SA-NSFC.

The ORR performance of the Fe-SA-NSFC in a 0.1 M HClO_4_ acid electrolyte solution reflected a significantly improved ORR activity in terms of a high *E*_1/2_ of 0.82 V compared with those of the Fe-SA-NC (*E*_1/2_ = 0.72 V) and Fe-SA-NSC (*E*_1/2_ = 0.79 V), and it was only 40 mV lower than that of the Pt/C catalyst (*E*_1/2_ = 0.86 V) (Fig. 4e and Supplementary Table 6). The *J*_k_ of Fe-SA-NSFC was as high as 1.25 mA cm^−2^ at 0.85 V, which was 4.0 and 3.0 times higher than those of the Fe-SA-NC (*J*_k_ = 0.31 mA cm^−2^) and Fe-SA-NSC (*J*_k_ = 0.41 mA cm^−2^). The ORR activity of the Fe-SA-NSFC was superior or comparable to those of most reported non-noble metal electrocatalysts, such as N-coordinated SA Mn sites on graphitic carbon (Mn-N-C) (*E*_1/2_ = 0.80 V)^31^, Fe-SAs/NPS-HC (*E*_1/2_ = 0.80 V)^42^, Fe-N-C catalysts prepared by ball-milling-assisted method (*E*_1/2_ = 0.81 V)^43,44^, and SA-Fe-N catalyst (*E*_1/2_ = 0.812 V)^42^ (Supplementary Tables 6 and 8). However, Fe-SA-NSFC exhibits a lower activity than some catalysts with much lower Fe loading such as Fe-doped ZIF-derived catalysts (*E*_1/2_ = 0.85 V, Fe loading: 0.45 at%)^27^ and ammonia-activated Fe-N-C (outperformed commercial Pt/C)^45^. Some Fe atoms that were trapped in the carbon layer probably could not contribute to ORR activity due to their inaccessibility. Therefore, the next challenge is proposed to enhance the accessibility of the single Fe atoms, while keeping the high loading.

In addition, the Tafel slope of the Fe-SA-NSFC was determined to be 57 mV dec^−1^, indicating that the Fe-SA-NSFC has the similar rate-determining step (RDS) of the ORR in the acidic electrolyte solutions (Supplementary Fig. 18a). The fast ORR kinetics of the Fe-SA-NSFC could favour H_2_O formation through the 4*e*^−^ pathway (*n* = 3.97 and H_2_O_2_ yield <1.7%) (Fig. 4f). In addition, the ADT result demonstrated that the *E*_1/2_ of Fe-SA-NSFC only decreased by 24 mV after 20,000 CV scans, again revealing a durability superior to that of commercial Pt/C (~63 mV loss) (Supplementary Fig. 18d, e). Turnover frequencies (TOFs), representing the intrinsic activity of a catalyst, can be calculated as the total number of electron transfers per second during ORR over the number of ORR active sites (for details see the Supplementary Information)^46^. The TOFs of Fe-SA-NSFC were 0.22 at 0.85 V in alkaline solution and 0.17 e s^−1^ site^−1^ at 0.8 V in acid solutions, which were in the range of similar activities reported in previous works, indicating again, that Fe-SA-NSFC is a promising catalyst material for ORR applications (Supplementary Table 9)^46,47^.

### Correlation of ORR activity with S, F-co-doped FeN4 active sites

To understand why additional doping of S and F could enhance the ORR activity of FeN_4_ sites, the Gibbs free energy changes of the 4e- ORR process were computed for the three structures of Fe-SA-NSFC, Fe-SA-NC and Fe-SA-NSC (Fig. 4g), which were constructed according to the XANES and XPS results, literature^46^, and geometrically optimized by a density functional-based tight binding method (http://www.dftb.org) (Supplementary Figs. 19–22, see the details in the Supplementary Information). The free energy diagram for *U* = 0 V (vs. RHE) and pH 13 inferred that the OH* reduction was the RDS for FeN_4_ active sites in both the Fe-SA-NC and Fe-SA-NSC with high free energy changes of +0.77 and +0.72 eV, respectively. In contrast, the free energy change for OH* reduction on the FeN_4_ active sites with S, F co-doping was significantly decreased to −0.05 eV (Fig. 4h), suggesting that the ORR process on the Fe-SA-NSFC was thermodynamically favoured compared to that on the Fe-SA-NC and Fe-SA-NSC^48^. Mulliken charge analysis revealed an increased positive charge density for Fe sites in the S, F-co-doped FeN_4_ centre over that for the S-doped FeN_4_ (Fe-SA-NSC) and FeN_4_ (Fe-SA-NC) (Fig. 4i and Supplementary Fig. 24). As the positively charged site was more unfavourable for adsorption of ORR intermediates, this result was also consistent with the enhanced ORR activity of the Fe-SA-NSFC^49^. Indeed, the calculated adsorption energy of OH* on the FeN_4_ active site was significantly increased from −0.31 eV for the Fe-SA-NC to +0.52 eV for the Fe-SA-NSFC (Fig. 4i and Supplementary Table 10). A volcano curve clearly demonstrated a relationship between the ORR activity and the adsorption energy of OH* (Supplementary Fig. 25), in which Fe-SA-NC appeared on the left side of the peak. The strong binding between the surface of the FeN_4_ active sites and oxygen slowed the desorption of OH*, which hindered the completion of the ORR process. With S, F co-doping, the increased positive adsorption energy of OH* on FeN_4_ active centres could result in fast desorption of OH* and regeneration of active sites, achieving an ORR performance for Fe-SA-NSFC that is close to the apex of the volcano^50^.

### Zn-air battery performance

**Fig. 5:**
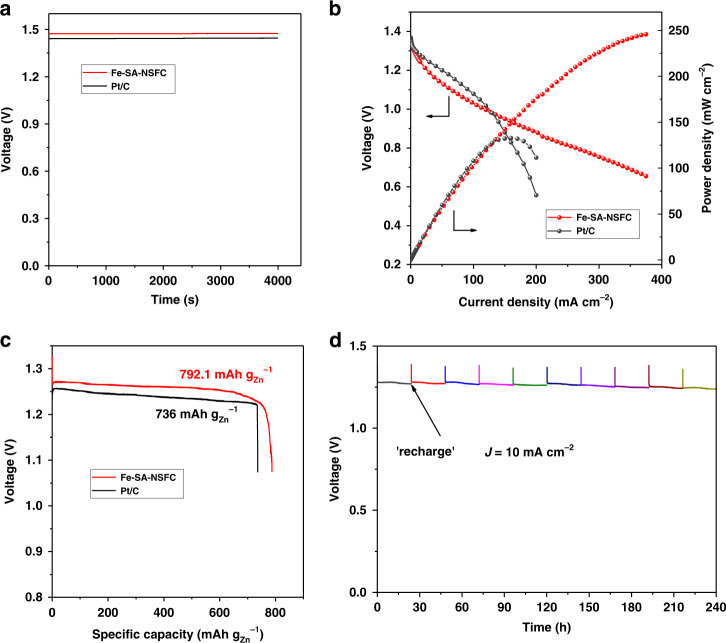
Performance of the Fe-SA-NSFC and Pt/C catalysts in a Zn-air battery. **a** The open-circuit voltage curves, **b** discharge polarization curves and corresponding power density plots, and **c** long-term galvanostatic discharge curves. The specific capacity was normalized to the mass of consumed Zn. **d** “Recharging” the Zn-air battery with Fe-SA-NSFC as the cathode catalyst by replenishing the Zn anode and electrolyte.

For practical application in energy devices, a primary Zn-air battery was assembled utilizing the Fe-SA-NSFC as an air electrode in a 6.0 M KOH electrolyte containing 0.2 M zinc acetate (Zn(OAc)_2_) (see the details in ‘Methods’). The Fe-SA-NSFC-based battery gave rise to a larger open-circuit voltage of 1.47 V than that of the Pt/C catalyst used as a reference (1.45 V) (Fig. 5a). The maximum power density of the Fe-SA-NSFC was as high as 247.7 mW cm^−2^ (Fig. 5b), which considerably outperformed those of the Pt/C catalyst (133 mW cm^−2^) and most state-of-the-art noble metal-free catalysts, such as Fe-SAs/NPS-HC (195.0 mW cm^−2^)^42^, atomic Co-N_*x*_-C (150 mW cm^−2^)^51^ and atomically dispersed Fe‐N_4_ in N‐doped porous carbon (Fe-N_4_ SAs/NPC) (232 mW cm^−2^)^52^ (Supplementary Table 11). Furthermore, the Fe-SA-NSFC-based Zn-air battery delivered a specific capacity of 792.1 mAh g_Zn_^−1^ at 10 mA cm^−2^, corresponding to ∼96.6% utilization of the theoretical capacity (∼820 mAh g_Zn_^−1^) (Fig. 5c)^18^. It worked stably in a mechanically rechargeable battery by only refuelling the consumed zinc anode and electrolytes at the end of each discharge. No noticeable degradation was observed after 10 cycles over a period of 240 h at a current density of 10 mA cm^−2^ (Fig. 5d). When being used as bifunctional oxygen electrocatalyst for a rechargeable Zn-air battery, Fe-SA-NSFC could also stably operate for 120 h at a constant charge–discharge current density of 10 mA cm^−2^ (Supplementary Fig. 26). The charge voltage was larger than 2.0 V as a result of the poor oxygen evolution reaction activity of Fe-SA-NSFC (Supplementary Fig. 27).

### Electrocatalytic HER performances of M-SA-NSFC (M = Co, Ru, Ir, and Pt)

The electrochemical HER performances of other M-SA-NSFC (M = Co, Ru, Ir, and Pt) SACs were investigated in a 0.5 M H_2_SO_4_ electrolyte solution. The Co-SA-NSFC exhibited an onset potential of 29 mV (Supplementary Fig. 28a). The overpotential was measured to be 104 mV at a current density of 10 mA cm^−2^, which was only ~71 mV lower than that of a benchmark Pt/C catalyst (32 mV at 10 mA cm^−2^). Moreover, this value was much lower than those for most previously reported Co-based HER electrocatalysts (Supplementary Table 12), such as Co and N co-doped mesoporous graphitic carbon catalysts (286 mV at 10 mA cm^−2^)^53^, a carbon supporting molecular CoN_*x*_ site catalyst (133 mV at 10 mA cm^−2^)^54^, Co_1_-N_4_/phosphorized carbon nitride (154 mV at 10 mA cm^−2^)^55^, and N-doped carbon nanotube hollow polyhedron supporting CoP nanoparticles (140 mV at 10 mA cm^−2^)^56^. The Tafel plot of Co-SA-NSFC provided further insight into the HER pathway. The Tafel slope of Co-SA-NSFC was ~66 mV dec^−1^ (Supplementary Fig. 28b), suggesting a Volmer–Heyrovsky mechanism for the Co-SA-NSFC catalyst and also indicating that the electrochemical H* desorption was the RDS^54^. The electrochemical HER performances of Ru-SA-NSFC, Ir-SA-NSFC, and Pt-SA-NSFC were also studied. The overpotentials at a current density of 10 mA cm^−2^ of Ru-SA-NSFC, Ir-SA-NSFC, and Pt-SA-NSFC were 62, 67 and 45 mV, respectively, implying comparable HER activities with reported noble metal-based electrocatalysts (Supplementary Table 12)^57–62^.

## Discussion

The key challenge of M-SACs has been a low metal loading, which has hampered both fundamental and practical applications. We demonstrated a versatile multilayer stabilization approach for fabricating high-loading M-SACs with powerful confinement and stabilization capacity towards metal species. A variety of non-precious and noble metals achieved atomic dispersion on graphitized carbon supports with a record metal loading of up to nearly 16 wt%. The optimized Fe-SAC, namely, the Fe-SA-NSFC, demonstrated a remarkable ORR performance and outperformed a commercial Pt/C catalyst in a Zn-air battery with regard to its long-term stability. Moreover, the optimized Co-SA-NSFC showed a good HER performance in acid media. Our method is very versatile in terms of metal species and the control of their loading. Therefore, it serves as a versatile platform to further explore the potential of high-loading M-SACs and their applications in energy devices and catalysis in other fields, including artificial photosynthesis and synthetic organic chemistry.

## Methods

### Preparation of the Fe-SA-NSFCs

Ferrocene (0.2 mmol), thiourea (0.7 mmol), and PFTA (0.07 mmol) were dissolved in ethanol (2 mL) and then water (10 mL) was added. After vigorous stirring for 10 min, pyrrole (124 μL) was added dropwise into the above solution with stirring for 20 min. The APS aqueous solution (0.18 M, 1 mL) was then added and the mixture was stirred for 10 s, and then left without stirring for 4 h to form a dark hydrogel. This hydrogel was then transferred into a Teflon-lined stainless-steel autoclave and heated at 150 °C for 8 h. The prepared hydrogel was freeze-dried and pyrolysed at 1000 °C for 1 h with a heating rate of 35 °C min^−1^ under flowing Ar (flow rate: 50 mL min^−1^). Fe-SA-NSFCs with different Fe loadings and Fe-NSFCs were fabricated by using 0.1, 0.15, and 0.25 mmol ferrocene, respectively.

### Preparation of the Fe-NP-NSC and Fe-NP-NSFC

Fe-NP-NSC was synthesized in the absence of PFTA, and Fe-NP-NSFC was fabricated using FeSO_4_·7H_2_O (0.2 mmol) as the Fe precursor. The experimental procedures were the same as for Fe-SA-NSFC.

### Preparation of the Fe-SA-NC and Fe-SA-NSC

Fe-SA-NC without S, F dopants and Fe-SA-NSC without F dopants were synthesized using a similar method described above. For the Fe-SA-NSC, the PFTA in the hydrogel formed after the hydrothermal reaction was removed by 1 M HCl ethanol solution and then rinsed with water three times^34^. Then, the hydrogel was freeze-dried and pyrolysed to obtain Fe-SA-NSC. The Fe-SA-NC was fabricated in the absence of thiourea. The APS and PFTA were washed out after polymerization of the Ppy for 4 h. The following procedures, including hydrothermal reaction and pyrolysis, were the same as mentioned above.

### Preparation of the M-SA-NSFCs (M = Co, Ru, Ir, and Pt)

These catalysts were synthesized using similar procedures, whereas cobaltocene (0.15 mmol), ruthenocene (0.15 mmol), (1,5-Cyclooctadiene)(methoxy)iridium(I) dimer (0.06 mmol), and platinum(II) acetylacetonate (0.10 mmol) were used as the corresponding metal precursors. Herein, another 0.8 mL of tetrahydrofuran was added to the ethanol to improve the solubility of the noble metal precursors. The same pyrolysis process as that used for the Fe-SA-NPSFC was used for the preparation of Co-SAC. For the noble metal SACs, the thermal reduction process was carried out at 250 °C for 2 h under a H_2_/Ar atmosphere (100 mL min^−1^ for H_2_ and 50 mL min^−1^ for Ar).

### Physical characterization

The crystalline phases in the samples were investigated by powder XRD with a Rigaku D/max2500 diffractometer (Cu*K*α_1_ radiation, *λ* = 1.54059 Å). The metal loadings were tested by ICP-MS on a VISTA MPX (Varian, Inc.). The porous structure was analysed with N_2_ adsorption–desorption experiments conducted at 77 K on a Quantachrome SI-MP Instrument. The morphology was characterized by field-emission SEM on a JSM-7800F operating at 15 kV and TEM on a JEOL-2100F. The HRTEM images and element mappings were collected using Tecnai G2 F30 S-Twin TEM (FEI, The Netherlands) operated at 200 kV. The HAADF-STEM images were obtained by using a JEOL JEM-ARM200F S/TEM with a spherical aberration corrector. The XAFS analysis of Fe K-edge, Co K-edge, Ru K-edge, Ir K-edge, and Pt L3-edge, and their corresponding references were obtained at the 1W1B station of the Beijing Synchrotron Radiation Facility that was operated at 2.5 GeV with a maximum current of 250 mA. The acquired EXAFS data were analysed according to the standard procedures using the ATHENA module implemented in the IFEFFIT software packages (see the details in Supplementary Materials)

### Electrochemical ORR measurements

A CHI 760E electrochemical station (CH Instruments, Inc., Shanghai) was employed to measure the electrochemical performance using a standard three-electrode system. A Pt wire was used as the counter electrode, a Hg/HgO (4.24 M KOH solution) electrode and an Ag/AgCl (4.0 M KCl) electrode were used as the reference electrodes in 0.1 M KOH and 0.1 M HClO_4_ electrolyte solutions, respectively. An RDE with a glassy carbon disk (5.0 mm diameter) and an RRDE electrode with a glassy carbon disk (5.61 mm diameter) and a Pt ring (6.25 mm inner diameter and 7.92 mm outer diameter) were utilized as the substrates for the working electrode. The catalytic ink was prepared by dispersing 2.5 mg catalyst powder in 500 µL of a mixture solution containing 490 mL of ethanol and 10 µL Nafion solution (5 wt%) followed by ultrasonication for 2 h. The ink was drop-cast on the disk electrode with a loading of 0.5 mg cm^−2^ to yield a uniform film electrode. CV was first carried out in O_2_-saturated electrolyte solution to activate the catalysts until the CV profile was stable. Then, the LSV polarization curves were recorded with a scan rate of 10 mV s^−1^ at different rotation speeds from 225 to 1600 rpm. The catalytic durability was investigated by an ADT test with CV under potentials from 0.6 to 1.0 V in the accelerated O_2_-saturated 0.1 M KOH or 0.1 M HClO_4_ electrolyte solution with a sweep rate of 50 mV s^−1^. The chronoamperometric measurement (*i*–*t*) was carried out at 0.9 V in 0.1 M KOH electrolyte solution that was pre-saturated with oxygen for 25,000 s with a rotation rate of 1600 r.p.m. A commercial Pt/C (20 wt% Pt, Fuelcellstore) was used for comparison with a Pt/C loading of 0.1 mg cm^−2^. The detailed analysis is provided in the Supplementary Materials.

### Electrochemical HER measurements

The electrochemical HER performance of the M-SA-NSFC (M = Co, Ru, Ir, and Pt) were evaluated using a RDE technique in an Ar-saturated 0.5 M H_2_SO_4_ solution. A graphite rod and an Ag/AgCl (4.0 M KCl) electrode were used as counter and reference electrode, respectively. The RDE with a catalyst loading of 0.5 mg cm^−2^ was used as working electrode. The HER polarization curves were collected from the potential range of 0 to approximately −0.25 V (vs. RHE) at a scan rate of 2 mV s^−1^ and an electrode rotation speed of 1600 r.p.m.

### Zn-air battery measurements

A home-built electrochemical cell was chosen to study the Zn-air battery performance of the Fe-SA-NSFC. The catalytic ink was loaded on carbon fibre paper (1 cm^2^) with a loading density of 1 mg cm^−2^. This carbon fibre paper and polished Zn foil were used as the air cathode and anode, respectively. A 6.0 M KOH aqueous solution containing 0.2 M Zn(OAc)_2_ was employed as the electrolyte solution. All data were recorded from this cell on a Land CT2001A system at room temperature.

### Computational methods

All theoretical calculations were performed using DFT with the CASTEP programme package in Materials Studio (version 2018), Accelrys, Inc. The Perdew–Burke–Ernzerhof (PBE) generalized gradient approximation (GGA) functional and ultrasoft pseudopotentials were employed to calculate the electronic exchange-correlation energies. The Grimme’s DFT-D correction was adopted for the van der Waals interactions because of the failure of GGA/PBE functional to describe nonlocal dispersion force. The cut-off energy of the plane wave basis set was chosen to be 400 eV, and a 4 × 4 × 1 Monkhorst-pack *k*-point grid over the Brillouin zone was used for the geometry optimization and energy calculations. The convergence criterion of energy was 10^−5^ Ha, the maximum force was 0.002 Ha Å^−1^ and the maximum displacement of 0.005 Å of was used for all cases. The details are provided in the Supplementary Materials.

## Supplementary information

Supplementary Information

## Data Availability

The data that support the plots within this paper and other findings of this study are available from the corresponding author on reasonable request.
