# Supplementary material for: Multilayer stabilization for fabricating high-loading single-atom catalysts
Source: Nat Commun. 2020 Nov 18;11:5892. doi: 10.1038/s41467-020-19599-8 (PMC7674447; doi:10.1038/s41467-020-19599-8)
Supplement: Supplementary file 1 — Supplementary Information [file 41467_2020_19599_MOESM1_ESM.pdf]

# Multilayer Stabilization for Fabricating High-Loading Single-Atom Catalysts

**Yazhou Zhou[1,2], Xiafang Tao[1,2], Guangbo Chen[3], Ruihu Lu[4], Ding Wang[1], Ming-Xi Chen[5], Enquan Jin[1], Juan Yang[2], Hai-Wei Liang[5], Yan Zhao[4], Xinliang Feng[3], Akimitsu Narita[1,6,]* & Klaus Müllen[1,]***

1. Max Planck Institute for Polymer Research, 55128 Mainz, Germany

2. School of Materials Science and Engineering, Jiangsu University, Zhenjiang, Jiangsu 212013, China

3. Center for Advancing Electronics Dresden (Cfaed) and Faculty of Chemistry and Food Chemistry Technische Universität Dresden 01062 Dresden, Germany

4. State Key Laboratory of Silicate Materials for Architectures, International School of Materials Science and Engineering, Wuhan University of Technology, Wuhan, Hubei 430070, China

5. Hefei National Laboratory for Physical Sciences at the Microscale, Department of Chemistry, University of Science and Technology of China, Hefei 230026, China

6. Organic and Carbon Nanomaterials Unit, Okinawa Institute of Science and Technology Graduate University, Okinawa 904-0495, Japan

## Supplementary Methods

**Extended X-ray absorption fine structure (EXAFS) analysis**: The acquired EXAFS data were obtained according to the standard procedures using the ATHENA module implemented into the IFEFFIT software packages. The EXAFS spectra were achieved by subtracting the post-edge background from the overall absorption followed by normalization with respect to the edge jump step. Then, χ(k) data in the k-space were Fourier transformed to real (R) space using hanning windows ($d_k$ = 1.0 $Å^{-1}$) to separate the EXAFS contributions from different coordination shells. The quantitative information could be obtained by the least-squares curve fitting in the R space with a Fourier transform k space, using the module ARTEMIS of programs of IFEFFIT. The backscattering amplitude F(k) and phase shift Φ(k) were calculated using FEFF8.0 code.

**Electrochemical measurements**:

All the potentials in this work were converted to reversible hydrogen electrode (RHE) according to:

$E$ ($vs$. RHE) = $E$ ($vs$. Ag/AgCl) + 0.261 V (1)

$E$ ($vs$. RHE) = $E$ ($vs$. Hg/HgO) + 0.878 V (2)

(1) 0.1 M $HClO_4$ and (2) 0.1 M KOH were used as electrolyte solutions, respectively.

The electron-transfer number (n) was calculated by the Koutecky-Levich (K-L) equation[1].

$$\frac{1}{J} = \frac{1}{J_L} + \frac{1}{J_K} = \frac{1}{B\omega^{\frac{1}{2}}} + \frac{1}{J_K} \quad (3)$$

$$B = 0.62nFC_0 D_0^{\frac{2}{3}} V^{-\frac{1}{6}} \quad (4)$$

where $J$ is the measured current density, $J_K$ and $J_L$ are the kinetic and limiting current densities, respectively, $\omega$ is the angular velocity of the disk, n is the overall number of electrons transferred in oxygen reduction, F is the Faraday constant (96485 C $mol^{-1}$), $C_0$ is the bulk concentration of $O_2$ (1.2 × $10^{-6}$ mol $cm^{-3}$ in 0.1 M KOH solution and 1.26 × $10^{-6}$ mol $cm^{-3}$ in 0.1 M $HClO_4$ solution, respectively), $D_0$ is the diffusion coefficient of $O_2$ (0.1 M KOH solution: 1.9 × $10^{-5}$ $cm^2$ $s^{-1}$, 0.1 M $HClO_4$ solution: 1.93 × $10^{-5}$ $cm^2$ $s^{-1}$), and V is the kinematic viscosity of the electrolyte (0.01 $cm^2$ $s^{-1}$) [2, 3].

The hydrogen peroxide yield ($H_2O_2$%) and the electron-transfer number (n) were determined by the rotating ring disk electrode (RRDE) technique. The ring potential was constant at 1.45 V vs. RHE. The equations were as follows:

$$H_2O_2\ (\%) = 200 \times \frac{\frac{I_r}{N}}{I_d + \frac{I_r}{N}} \quad (5)$$

$$n = 4 \times \frac{I_D}{\frac{I_R}{N} + I_D} \quad (6)$$

where $I_D$ is the disk current, $I_R$ is the ring current, and N = 0.37 is the collection efficiency of Pt ring.

The turnover frequencies (TOFs) of the catalysts were calculated according to the following equation[4]:

$$\text{TOF} = \frac{J_k N_e}{\omega_{Fe} c_{cat} N_A / M_{Fe}} \quad (7)$$

where $J_k$ is the kinetic current density (mA $cm^{-2}$), $N_e$ represents the electron number per Coulomb $6.24 \times 10^{18}$, $\omega_{Fe}$ is the atomic content of active Fe in Fe-SA-NSFC (it is considered that the surface Fe atoms are the exposed active sites of the catalyst, thus the $\omega_{Fe}$ value has been determined from XPS data), $c_{cat}$ is the catalyst loading, $N_A$ is the Avogadro constant $6.022 \times 10^{23}$, and $M_{Fe}$ is the molar mass of Fe (55.845 g $mol^{-1}$).

**Computational details**.

**Structures.** The structures for Fe-SA-NSFC, Fe-SA-NC and Fe-SA-NSC were constructed according to the XANES and XPS results, as well as the literature work[4]. A density functional-based tight binding (DFTB) method was employed to geometrically optimize the proposed structures [5]. Their procedures were shown in pages of 24-26. DFTB enables one to perform faster geometry optimization calculations of larger as well as periodic systems compared with DFT (http://www.dftb.org). DFTB geometry optimized results can be used as a fast pre-optimizer for further periodic DFT calculations.[6, 7]

**Adsorption energy.** The adsorption energy change ($\triangle E_{ads}$) of the key ORR intermediates, including *OOH, *O and *OH, was calculated relative to $H_2O$ and $H_2$ under conditions of $T$ = 298.15 K, pH = 0 and $U$ = 0 V (*vs*. RHE) according to the following equations[8]:

$$\triangle E_{*OOH} = E_{*OOH} + 3/2\, E_{H2} - E_{*} - 2\, E_{H2O} \quad (8)$$

$$\triangle E_{*O} = E_{*O} + E_{H2} - E_{*} - E_{H2O} \quad (9)$$

$$\triangle E_{*OH} = E_{*OH} + 1/2\, E_{H2} - E_{*} - E_{H2O} \quad (10)$$

where * represented the adsorption sites associated with $FeN_4$ doped graphene. The $\triangle E_{ads}$ was defined as the adsorption energy change of the following steps.

$$* + 2H_2O \rightarrow *OOH + 3/2H_2 \quad (11)$$

$$* + H_2O \rightarrow *O + H_2 \quad (12)$$

$$* + H_2O \rightarrow *OH + 1/2H_2 \quad (13)$$

**The Gibbs free energy variation.** Gibbs free energy change ($\triangle G$) of each adsorbed intermediate was calculated based on the computational hydrogen electrode method developed by Nørskov et al[9]. At standard condition ($T$ = 298.15 K, pH = 0, and $U$ = 0 V (vs. RHE)), $\triangle G$ was defined as the following equation:[9]

$$\triangle G = \triangle E + \triangle E_{ZPE} - T\triangle S + \triangle G_{pH} + \triangle G_{U} \quad (14)$$

where $\triangle E$ was the energy change obtained from DFT calculation, $\triangle E_{ZPE}$ was the difference between the adsorbed state and gas phase, which was calculated by summing vibrational frequency for all model based on the equation: $E_{ZPE} = 1/2\sum hV_i$ in the above reaction system. $T$ was the temperature = 298.15 K. $\triangle S$ represented the difference of the entropies between the adsorbed state and gas phase. The entropies of free molecules were obtained from NIST database (https://janaf.nist.gov/). $\triangle G_{pH} = -kT\ln[H^+] = pH\cdot kT\cdot \ln 10 = -0.0591pH$ was used to correct the free energy of $H^+ + e^-$ referenced by a RHE at various pH values. $\triangle G_U = -neU$, where $U$ was the applied electrode potential and $n$ was the number of transferring electrons. Hence, the equilibrium potential $U^0$ for ORR at pH = 13 was determined to be 0.462 V (*vs.* RHE).

The free energy of $O_2$ ($g$) was derived as $G_{O2(g)} = 2G_{H2O(l)} - 2G_{H2} + 4.92$ eV, and the free energy of $OH^-$ was calculated by $G_{OH-} = G_{H2O(l)} - G_{H+}$, where the energy of $H^+$ was approximately equal to the energy of $1/2H_2$. The overall reaction of $O_2$ reduction to $OH^-$ in an alkaline environment was: $O_2 + 2H_2O + 4e^- \rightarrow 4OH^-$, which was divided into four fundamental steps as follows：

(1) $O_2 (g) + H_2O (l) + e^- + * \rightarrow OOH* + OH^-$ (15)

(2) $*OOH + e^- \rightarrow O* + OH^-$ (16)

(3) $O* + H_2O (l) + e^- \rightarrow OH* + OH^-$ (17)

(4) $OH* + e^- \rightarrow * + OH^-$ (18)

* represented the adsorption site.

**Theoretical ORR Overpotential.** The theoretical ORR overpotentials ($\eta_{RHE}$, *vs.* RHE) associated with different active sites were calculated according to following equation:

$$H_{RHE} = 0.462V + \Delta G_{max} \quad (19)$$

where 0.462 V was defined as the equilibrium potential of the overall 4-electron ORR at the standard state and $\triangle G_{max}$ represented the most positive free energy variation associated with the proton-electron-transfer steps.

## Supplementary Figure 1-28 and Table 1-19.

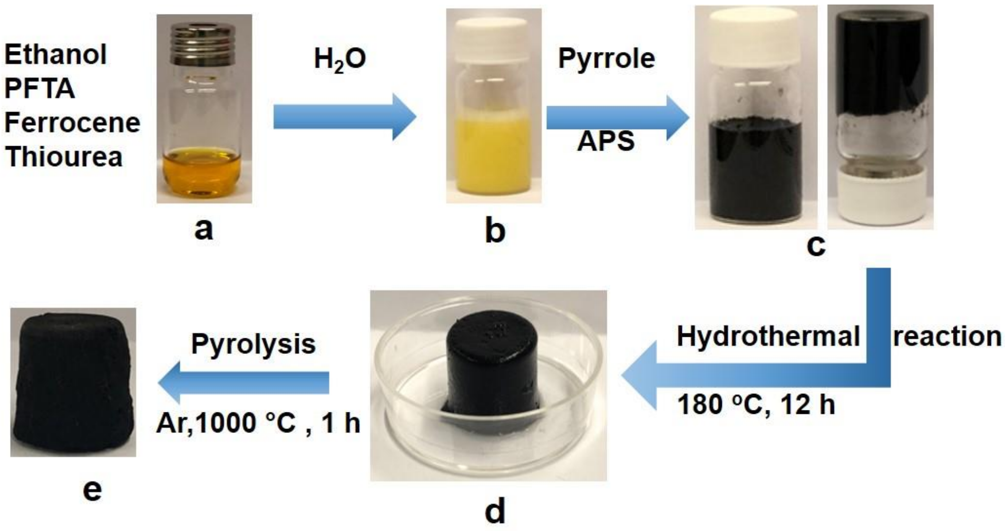


**Supplementary Figure 1. Digital photos of reaction status for different procedures**. **(a)** dissolving PFTA, ferrocene and thiourea into ethanol, **(b)** formation of PFTA/ferrocene/PFTA layers after adding water, **(c)** oxidative polymerization of pyrrole with ammonium persulfate (APS) to form Ppy(PFTA/ferrocene/PFTA), **(d)** establishing a free-standing hydrogel of Ppy(PFTA/ferrocene/PFTA) after hydrothermal reaction, **(e)** obtained Fe-SA-NSFPC after pyrolysis.

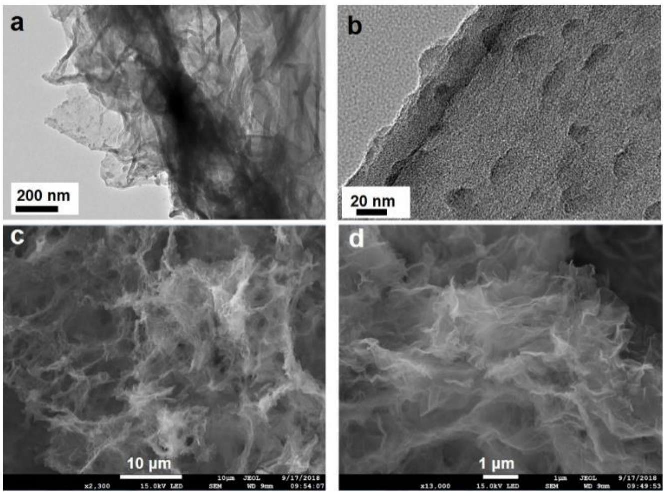


**Supplementary Figure 2**. **Morphology characterization**. **(a, b)** TEM images of Ppy(PFTA/ferrocene/PFTA) after polymerization of pyrrole and **(c, d)** SEM images of Fe-SA-NSFC.

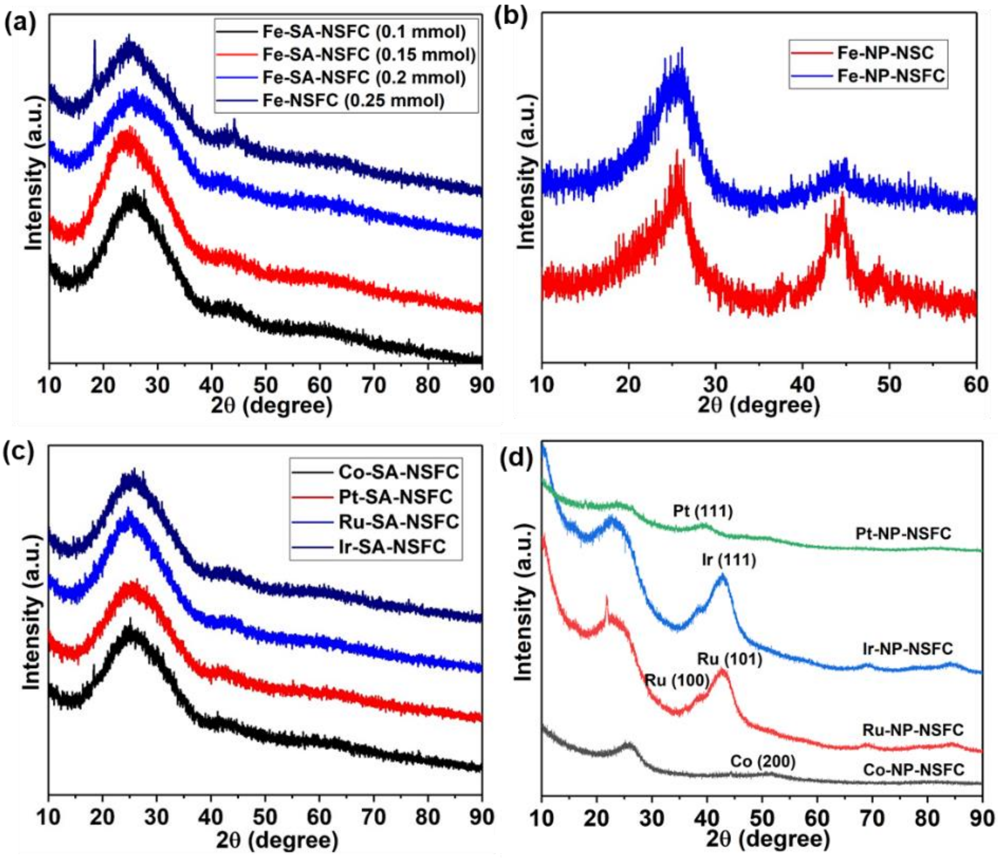


**Supplementary Figure 3**. **XRD analysis of samples**. **(a)** XRD patterns of Fe-SA-NSFC (4.6 wt%), Fe-SA-NSFC (8.7 wt%), Fe-SA-NSFC (15.3 wt%) and Fe-NSFC, **(b)** XRD patterns of Fe-NP-NSC and Fe-NP-NSFC reference samples prepared using similar procedures as for Fe-SA-NSFC, where Fe-NP-NSC was synthesized in the absence of PFTA and Fe-NP-NSC was prepared with $FeSO_4 \cdot 7H_2O$, **(c)** XRD patterns of M-SA-NSFC (M = Co, Pt, Ru and Ir) using corresponding organometallic precursors (0.15 mmol for Co, Ru, Ir-containing precursors and 0.10 mmol for Pt precursor). **(d)** XRD patterns of Co, Pt, Ru and Ir-containing samples prepared using a higher amount of metal precursors (0.20 mmol for Co, Ru precursors, 0.1 mmol for Ir precursors and 0.15 mmol for Pt precursor). As shown in panel d, the crystalline metal-based phase was detected for each metal, indicating that these amounts of organometallic precursors result in formation of metal aggregates.

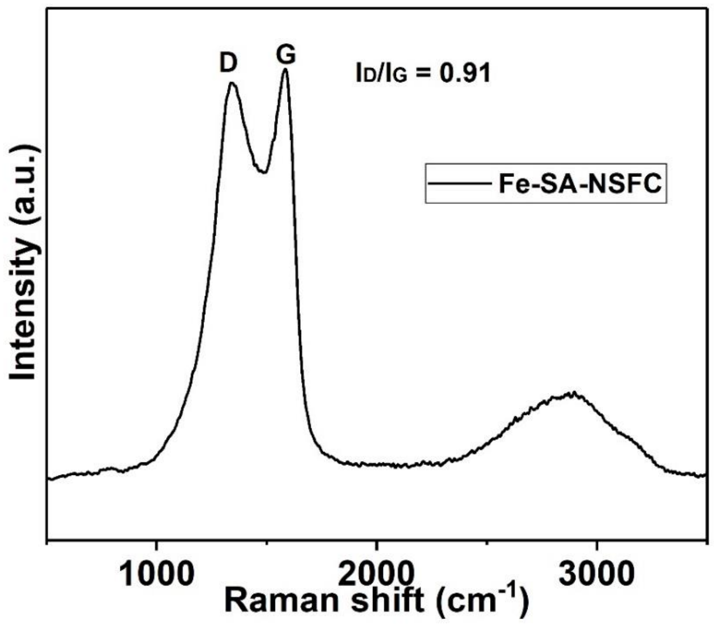


**Supplementary Figure 4.** Raman spectrum of Fe-SA-NSFC.

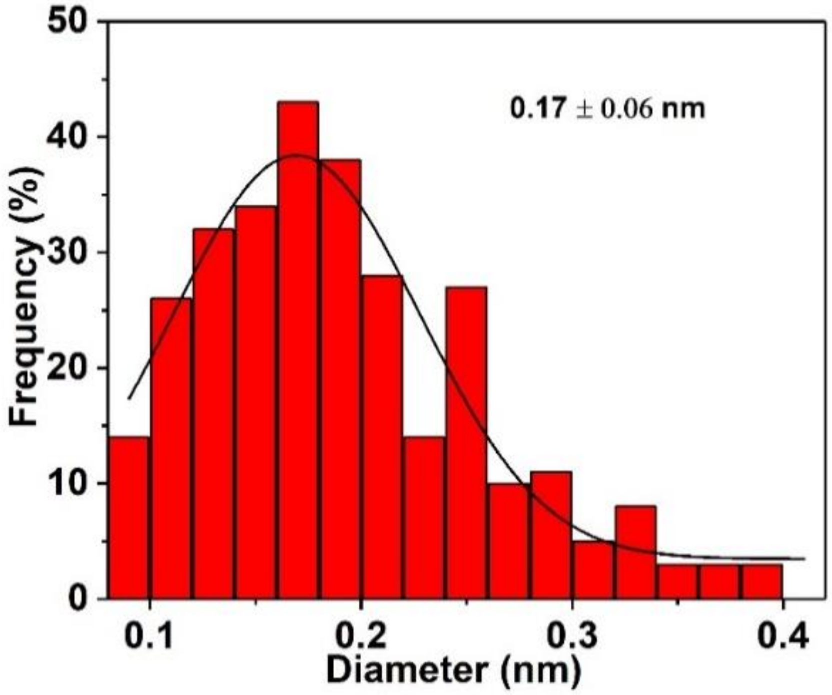


**Supplementary Figure 5**. **Size distribution of bright spots**. It is corresponding to Fe atoms for Fe-SA-NSFC based on statistical analysis over 500 bright spots in the AC-HAADF-STEM image (Fig. 1g) using ImageJ program.

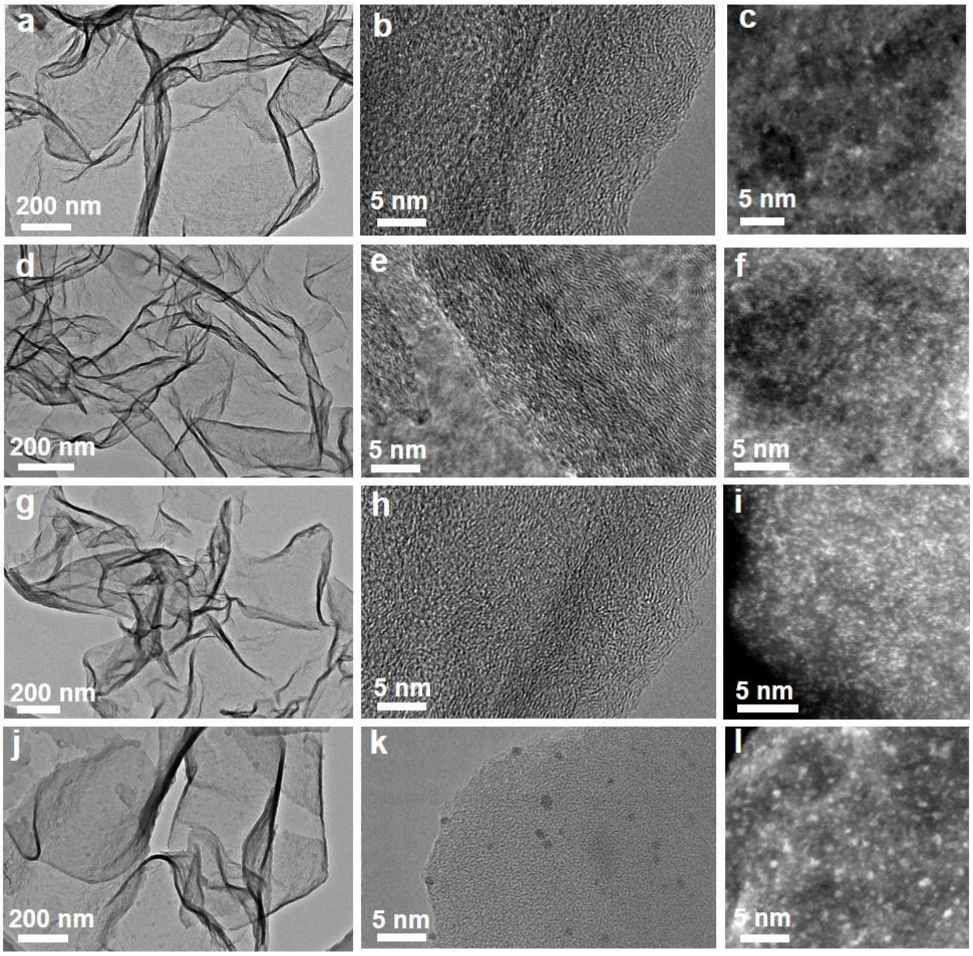


**Supplementary Figure 6**. **Morphology characterization of samples**. **(a, d, g, j)** TEM, **(b, e, h, k)** HRTEM, and **(c, f, i, l)** AC-HAADF-STEM images: **(a-c)** Fe-SA-NSFC (0.1 mmol), **(d-f)** Fe-SA-NSFC (0.15 mmol) **(g-i)** Fe-SA-NSFC (0.2 mmol) and **(j-l)** Fe-NSFC (0.25 mmol).

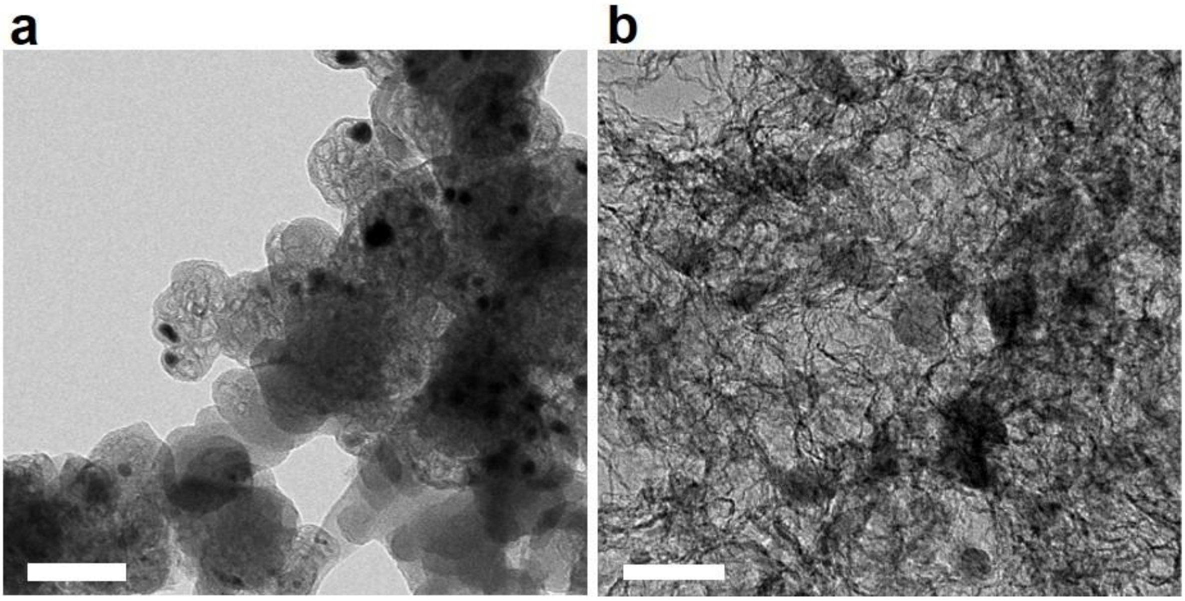


**Supplementary Figure 7**. **Morphology characterization of samples.** TEM images of **(a)** Fe-NP-NSC prepared in the absence of PFTA and **(b)** Fe-NP-NSFC fabricated using $FeSO_4 \cdot 7H_2O$ (0.2 mmol) as Fe precursor. Scale bar: 100 nm.

**Supplementary Table 1**. The metal loading of M-NSFCs (M = Fe, Co, Pt, Ru and Ir) catalysts from ICP-MS results as compared with literature data.

| Catalyst | Metal loading (wt%) | Ref. |
|---|---|---|
| Fe-SA-NSFC (0.1 mmol) | 4.8 | This work |
| Fe-SA-NSFC (0.15 mmol) | 8.6 | This work |
| Fe-SA-NSFC (0.2 mmol) | 15.3 | This work |
| Co-SA-NSFC | 12.2 | This work |
| Pt-SA-NSFC | 15.6 | This work |
| Ru-SA-NSFC | 10.9 | This work |
| Ir-SA-NSFC | 15.7 | This work |
| Pt/meso_S-C | 10 | 10 |
| Pd/meso_S-C | 5 | 4 |
| Rh/meso_S-C | 5 | 4 |
| Ru/meso_S-C | 3 | 4 |
| Ir/meso_S-C | 10 | 4 |
| 20Mn-NC-second | 3.03 | 11 |
| CoSSPIL/CNT | 4.0 | 12 |
| FeN4/GN | 4.0 | 13 |
| $FeC_{11}N_4$/CNS | 1.5 | 14 |
| SA-Fe-NHPC | 1.25 | 15 |
| Co–N–C@F127 | 6.2 | 16 |
| Pt-ISAS@NaY | 0.22 | 17 |
| Pd-ISAS@NaY | 0.56 | 11 |

| | | |
|---|---|---|
| Ru-ISAS@NaY | 0.22 | 11 |
| Rh-ISAS@NaY | 0.09 | 11 |
| Co-ISAS@NaY | 0.65 | 11 |
| Ni-ISAS@NaY | 0.31 | 11 |
| Cu-ISAS@NaY | 0.29 | 11 |
| Fe–NC SAC | 8.9 | 18 |
| Co-NC SAC | 12.1 | 12 |
| Pt/HSC | 5 | 19 |
| PtSA-NT-NF | 1.76 | 20 |
| Pt/$MoS_2$ | 7.5 | 21 |
| FePt NSs | 6.7 | 22 |
| CoSAs/N-C(800) | 4 | 23 |
| Ir/$MgAl_2O_4$ | 0.2 | 24 |

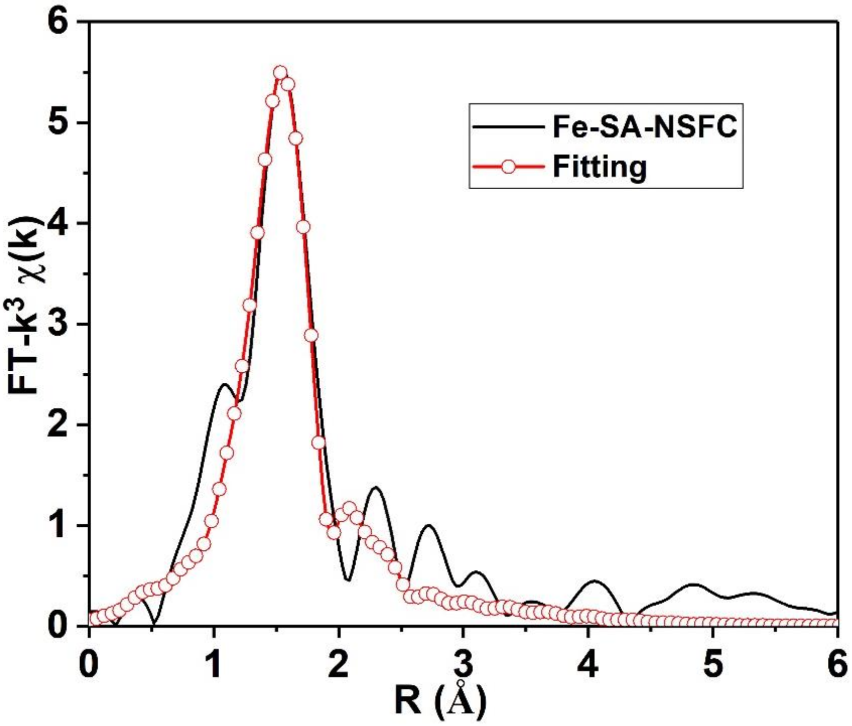


**Supplementary Figure 8. Fourier transforms of Fe K-edge EXAFS** (solid line) and corresponding fitting (open circle) curves of Fe-SA-NSFC.

**Supplementary Table 2.** Structural parameters of M-SA-NSFCs (M = Fe, Co, Ru, Ir and Pt) extracted from the EXAFS fitting ($S_0^2$=0.85).

| Catalyst | Shell | CN | $R_j$ (Å) | $\sigma^2$ ($10^{-3}$Å$^2$) | $\Delta E_0$ (eV) | R factor |
|---|---|---|---|---|---|---|
| Fe-SA-NSFC | Fe-N | 4.4 ± 0.5 | 1.97 ± 0.01 | 10.5 ± 2.3 | 9.3 ± 1.2 | 0.015 |
| Co-SA-NSFC | Co-N | 4.8 ± 1.6 | 1.87 ± 0.02 | 7.8 ± 2.5 | 6.0 ± 3.3 | 0.020 |
| Ru-SA-NSFC | Ru–N | 5.0 ± 0.6 | 2.07±0.02 | 6.9 ± 1.3 | 4.8 ± 0.5 | 0.020 |
| Ir-SA-NSFC | Ir-N | 4.0 ± 0.8 | 2.11 ± 0.02 | 7.1 ± 3.8 | 15.1± 2.2 | 0.026 |
| Pt-SA-NSFC | Pt-N | 4.0 ± 0.6 | 2.05 ± 0.02 | 5.0 ± 0.3 | 4.6 ± 1.4 | 0.020 |

$S_0^2$ is the amplitude reduction factor; CN is the coordination number; R is interatomic distance (the bond length between central atoms and surrounding coordination atoms); $\sigma^2$ is Debye-Waller factor (a measure of thermal and static disorder in absorber-scatter distances); $\Delta E_0$ is edge-energy shift (the difference between the zero kinetic energy value of the sample and that of the theoretical model). R factor is used to evaluate the goodness of the fitting.

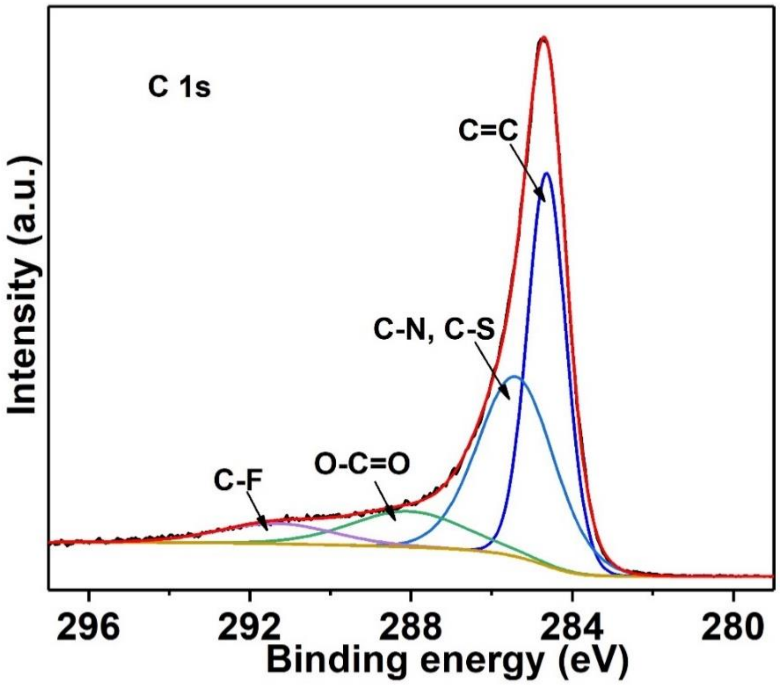


**Supplementary Figure 9.** C 1s XPS spectrum of Fe-SA-NSFC.

**Supplementary Table 3.** Elemental compositions of Fe-SA-NSFC, Fe-SA-NSC and Fe-SA-NC according to XPS measurements.

| Catalyst | C (at%) | N (at%) | O (at%) | S (at%) | F (at%) |
|---|---|---|---|---|---|
| Fe-SA-NSFC | 72.0 | 17.2 | 4.6 | 2.5 | 2.2 |
| Fe-SA-NSC | 74.5 | 17.3 | 4.1 | 2.7 | / |
| Fe-SA-NC | 76.9 | 17.2 | 4.6 | / | / |

**Supplementary Table 4.** Fitting results for C 1s spectra of Fe-SA-NSFC, Fe-SA-NSC and Fe-SA-NC.

| Catalyst | C=C (at%) | C-N, C-S (at%) | O=C-O (at%) | C-F (at%) |
|---|---|---|---|---|
| Binding energy | 284.7 (eV) | 285.8 (eV) | 288.6 (eV) | 291.0 (eV) |
| Fe-SA-NSFC | 43.3 | 38.2 | 11.8 | 6.7 |
| Fe-SA-NSC | 52.4 | 35.0 | 12.6 | / |
| Fe-SA-NC | 53.4 | 32.8 | 13.8 | / |

**Supplementary Table 5.** Fitting results for N 1s spectra of Fe-SA-NSFC, Fe-SA-NSC and Fe-SA-NC.

| Catalyst | Pyridinic N (at%) | Graphitic N (at%) | $Fe-N_4$ (at%) | $NO_x$ (at%) |
|---|---|---|---|---|
| Binding energy | 398.6 (eV) | 400.8 (eV) | 399.5 (eV) | 402.5 (eV) |
| Fe-SA-NSFC | 22.1 | 54.2 | 15.0 | 8.7 |
| Fe-SA-NSC | 21.9 | 52.9 | 14.4 | 10.8 |
| Fe-SA-NC | 23.3 | 40.4 | 13.2 | 23.1 |

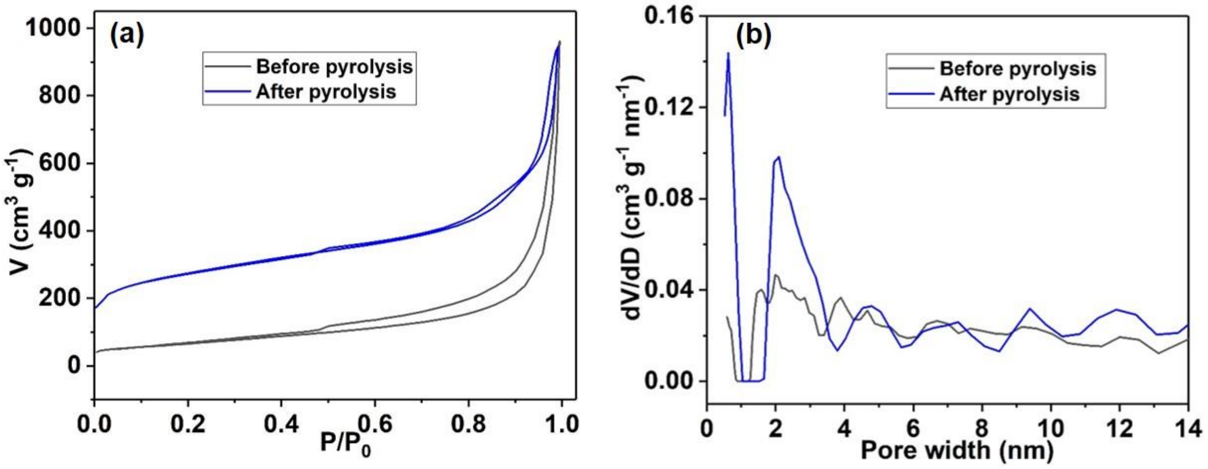


**Supplementary Figure 10. Porous characterization of samples. (a)** $N_2$ adsorption/desorption isotherms and **(b)** corresponding pore size distribution curve calculated from the adsorption branch for Fe-SA-NSFC before and after pyrolysis. Before measurements, all samples were degassed at 200 °C for at least 6 h. The pore size distribution curves were calculated by the nonlocal density functional theory (NDFT).

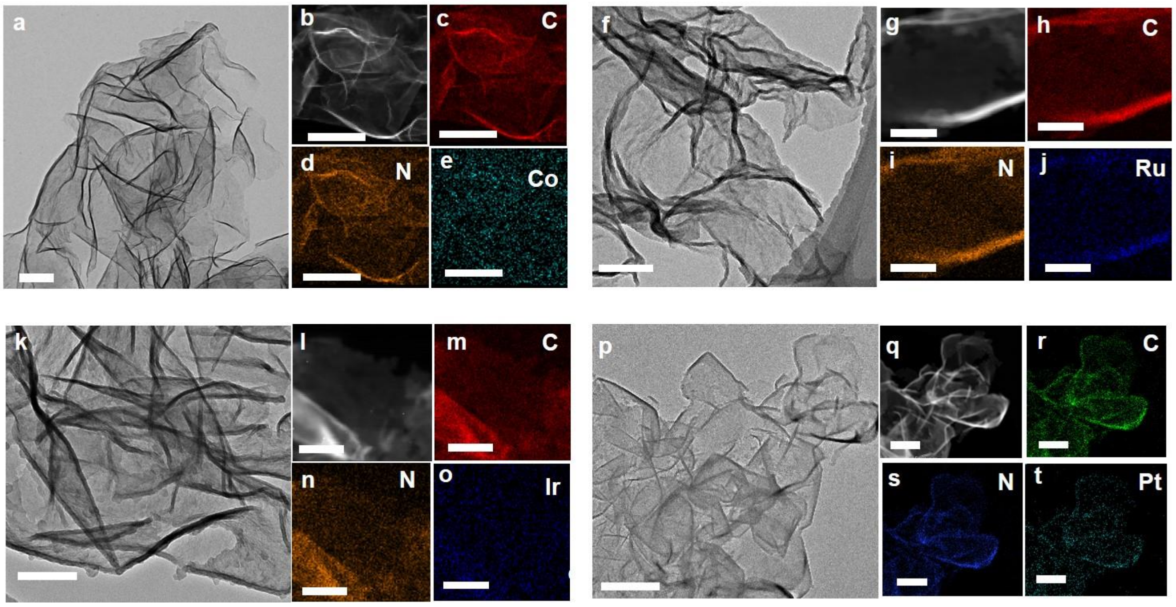


**Supplementary Figure 11**. **Morphology characterization of samples**. **(a, f, k, p)** TEM, **(b, g, l, q)** HAADF-STEM images and corresponding elemental mapping images: **(a-e)** Co-SA-NSFC, **(f-j)** Ru-SA-NSFC, **(k-o)** Ir-SA-NSFC and **(p-t)** Pt-SA-NSFC. Scale bars: 200 nm.

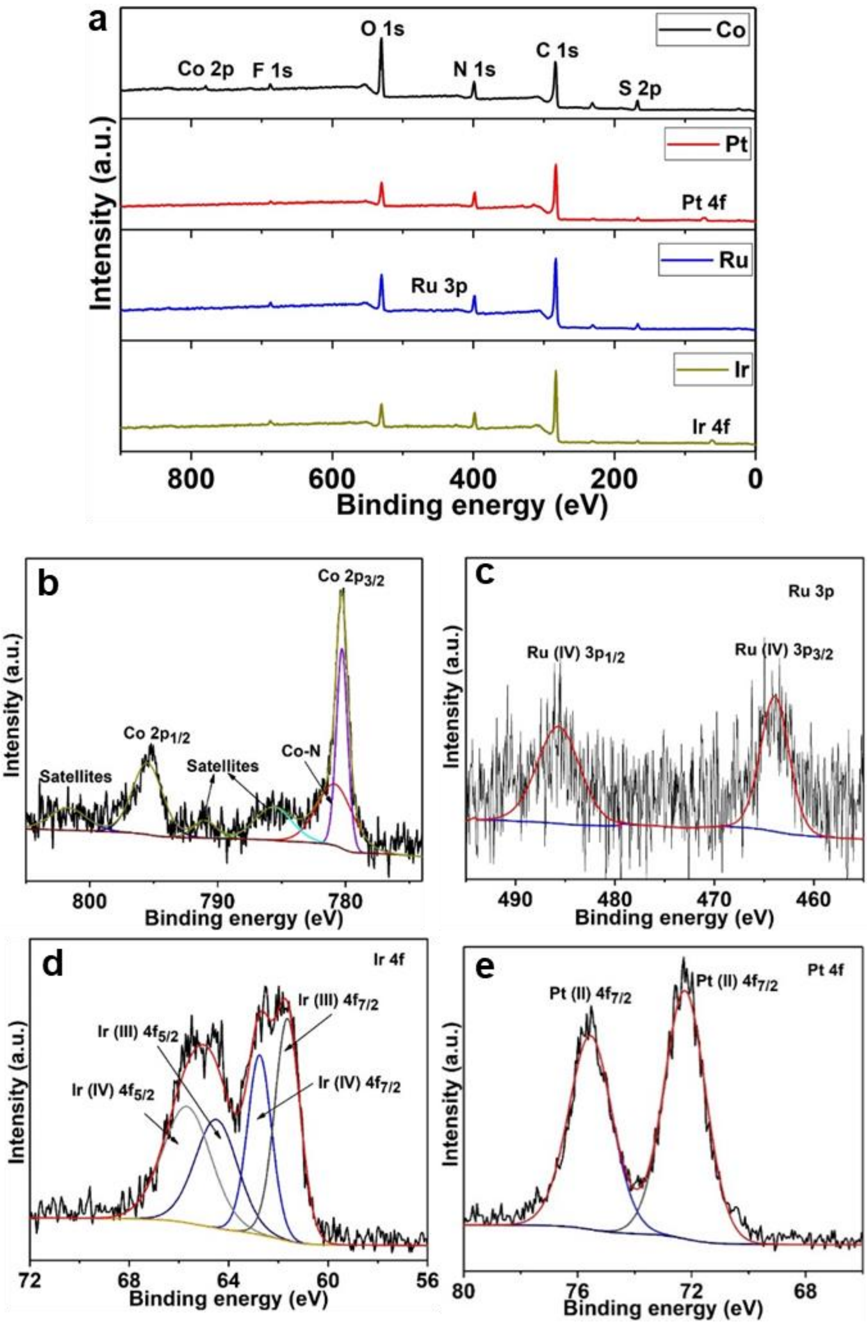


**Supplementary Figure 12. XPS analysis. (a) Survey XPS spectra of Co-SA-NSFC, Pt-SA-NSFC, Ru-SA-NSFC and Ir-SA-NSFC, (b)** Co 2p XPS spectrum of Co-SA-NSFC, **(c)** Ru 3p XPS spectrum of Ru-SA-NSFC, **(d)** Ir 4f XPS spectrum of Ir-SA-NSFC and **(e)** Pt 4f XPS spectrum of Pt-SA-NSFC.

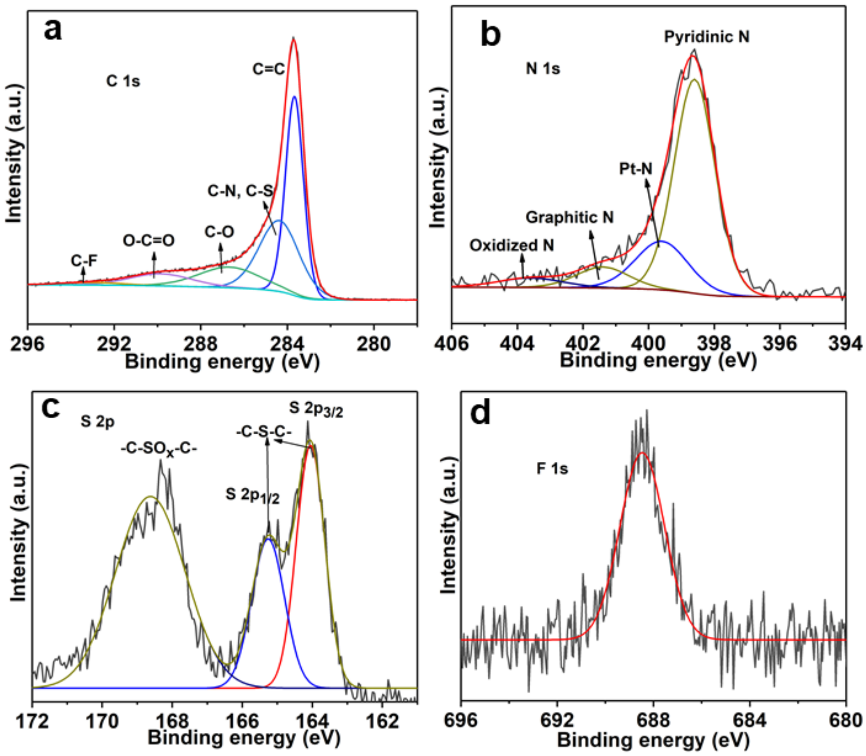


**Supplementary Figure 13. XPS analysis. (a)** C 1s, **(b)** N 1s, **(c)** S 2p and **(d)** F 1s XPS spectra of Pt-SA-NSFC. The XPS results revealed the heteroatoms doping structure in Pt-SA-NSFC, particularly O, N and S. The structure was different from that of Fe-SA-NSFC due to the low annealing temperature (250 °C). The Pt-SA-NSFC has higher oxygen content (~12.7 at%) than that of Fe-SA-NSFC (~4.6 at%). Pyridinic N was the main nitrogen species and graphitic nitrogen content in Pt-SA-NSFC was very low. The graphitic N was the dominant species in Fe-SA-NSFC as a result of the high temperature graphitization process. The S 2p XPS spectrum shows the coexistence of -C-$SO_x$-C- and –C-S-C- bonds in Pt-SA-NSFC. However, the weight of the -C-$SO_x$-C- bond in Fe-SA-NSFC is negligible.

.

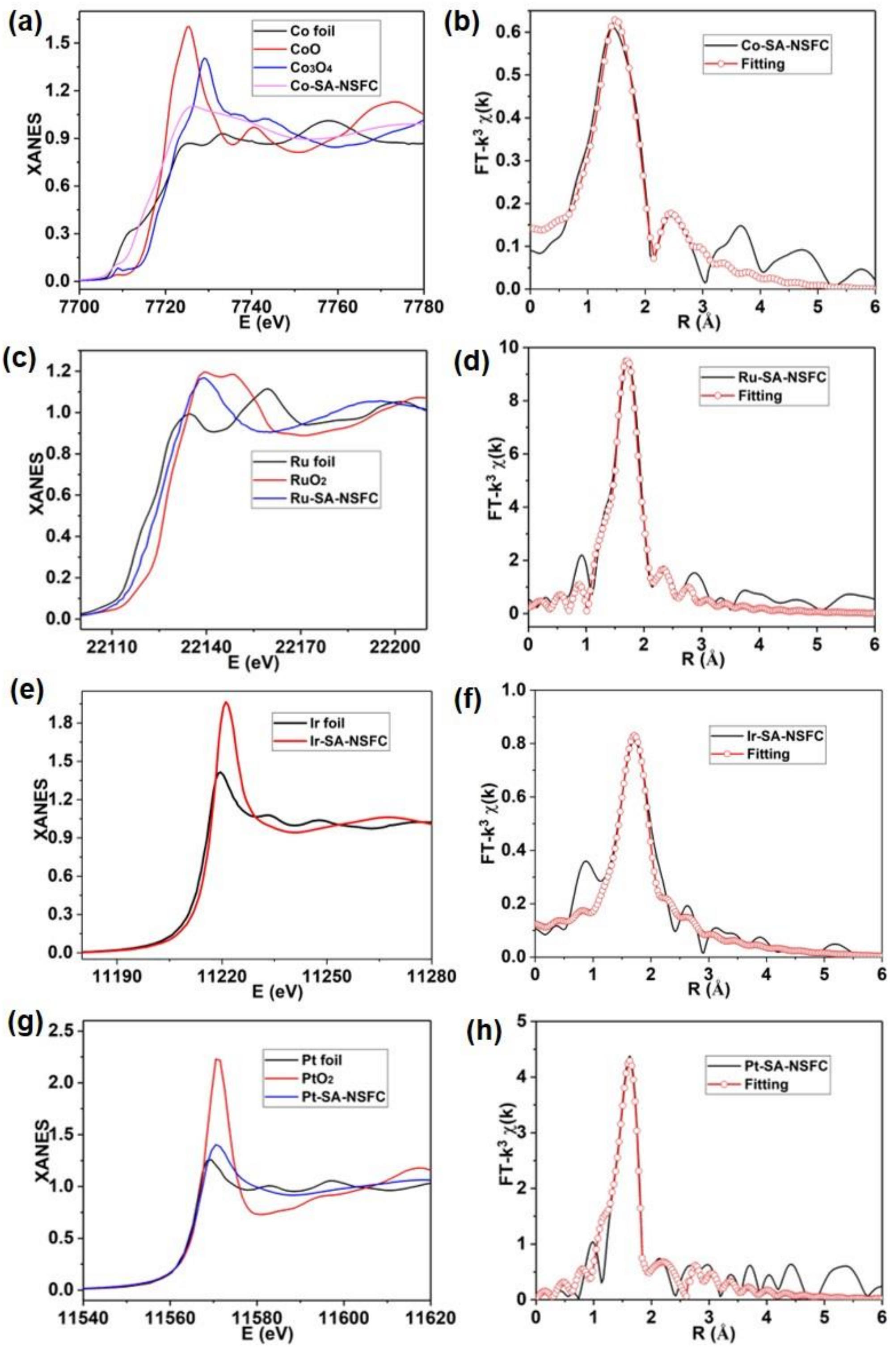


**Supplementary Figure 14**. **XAS analysis. (a, c, e, g)** XANES and **(b, d, f, h)** EXAFS fitting curves: **(a, b)** Co K-edge for Co-SA-NSFC, **(c, d)** Ru K-edge for Ru-SA-NSFC, **(e, f)** Ir $L_3$-edge for Ir-SA-NSFC, and **(g, h)** Pt $L_3$-edge for Pt-SA-NSFC at *R* space.

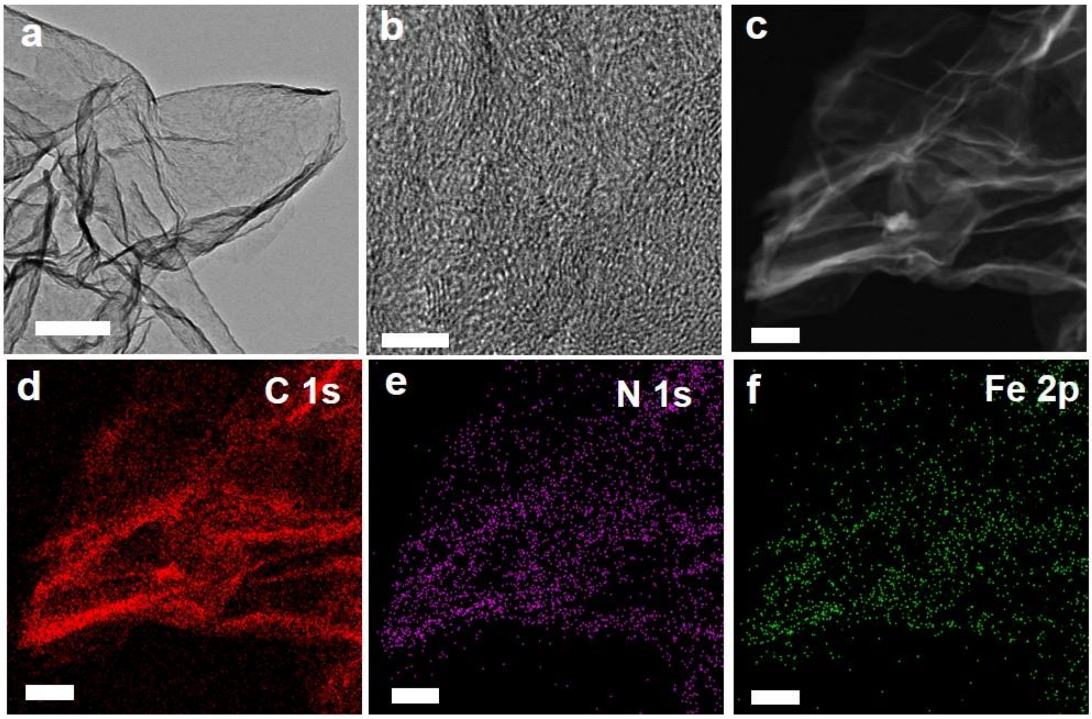


**Supplementary Figure 15. Morphology characterization of Fe-SA-NC. (a)** TEM, (**b)** HRTEM, **(c)** HAADF-STEM images of Fe-SA-NC and elemental mapping images of **(d)** C 1s, **(e)** N 1s and **(f)** Fe 2p. Scale bars in a, c, d, e, f: 200 nm, and scale bar in b: 10 nm.

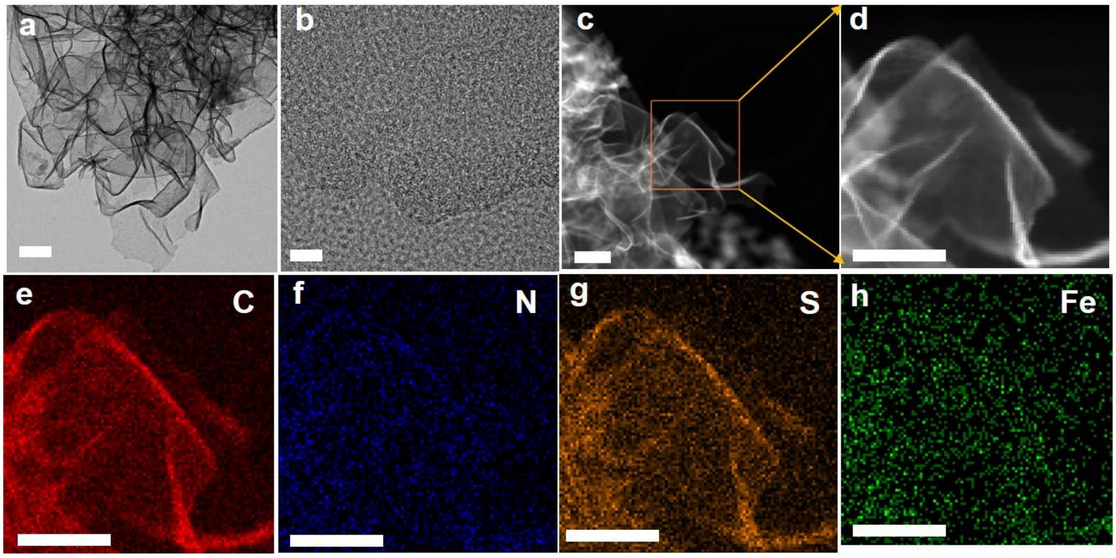


**Supplementary Figure 16. Morphology characterization of Fe-SA-NSC. (a)** TEM, **(b)** HRTEM, **(c)** HAADF-STEM images of Fe-SA-NSC, **(d)** enlarged HAADF-STEM image in d and elemental mapping images of **(e)** C 1s, **(f)** N 1s, **(g)** S 2p and **(h)** Fe 2p. Scale bars in a, c, e, f, g, h: 200 nm, and scale bar in b: 10 nm.

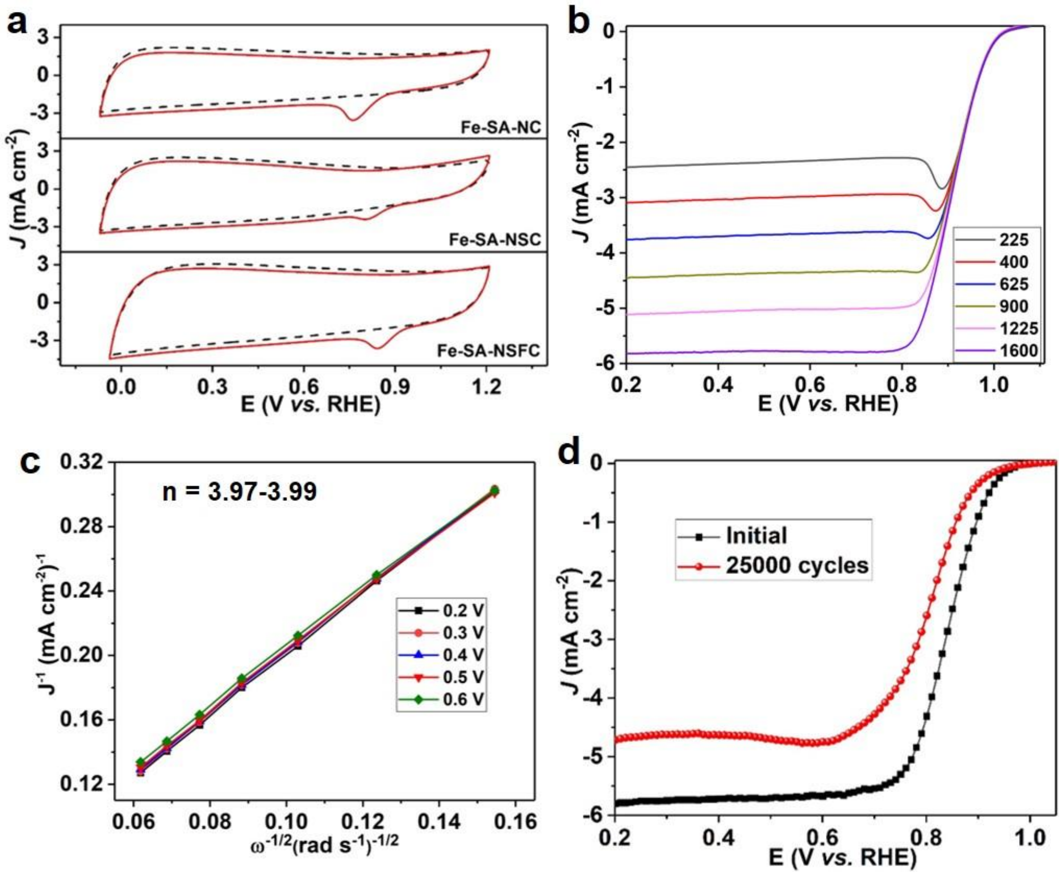


**Supplementary Figure 17. Electrochemical performances of catalysts in a 0.1 M KOH electrolyte solution. (a)** CV curves of Fe-SA-NC, Fe-SA-NSC and Fe-SA-NSFC, **(b)** ORR polarization curves of Fe-SA-NSFC at different rotation rates. A current peak was observed at ca. 0.9 V vs RHE using low rotation rates, which could be ascribed to a rapid decrease of oxygen concentration near the active sites as a result of insufficient oxygen transport at lower electrode rotation rates. This peak disappeared with an increasing rotation speed, suggesting that the oxygen concentration reached a dynamic equilibrium[25-27]. **(c)** K-L plots and electron-transfer numbers of Fe-SA-NSFC, **(d)** ORR polarization curves of commercial Pt/C (20 wt% Pt, Fuelcellstore, a loading of 0.1 mg $cm^{-2}$ on RDE) before and after 25000 CV cycles.

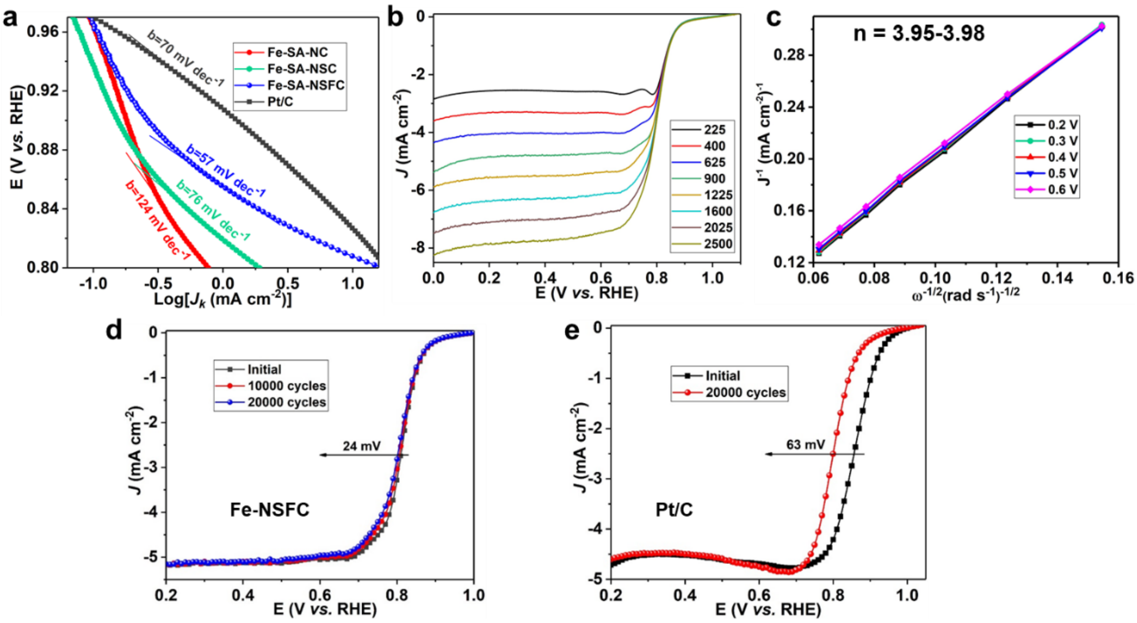


**Supplementary Figure 18. Electrochemical performances of Fe-SA-NC, Fe-SA-NSC, Fe-SA-NSFC and Pt/C catalysts in a 0.1 M $HClO_4$ electrolyte solution. (a)** Tafel plots, **(b)** ORR polarization curves of Fe-SA-NSFC at different rotation speeds, **(c)** K-L plots and electron-transfer numbers of Fe-SA-NSFC. ORR polarization curves of **(d)** Fe-SA-NSFC and **(e)** commercial Pt/C catalyst before and after ADTs.

**Supplementary Table 6.** Summary of ORR activity for the catalysts in 0.1 M KOH and 0.1 $HClO_4$ electrolyte solutions.

| Catalysts | Onset potential (V) | $E_{1/2}$ (V) | $J_d$ (mA cm$^{-2}$) | $J_k$ (mA cm$^{-2}$, at 0.85 V) | Tafel slope (mV decade$^{-1}$) | Electrolyte |
|---|---|---|---|---|---|---|
| Fe-SA-NSFC | 1.01 | 0.91 | 5.89 | 61.50 | 53 | 0.1 |
| Fe-NSFC | 0.97 | 0.85 | 4.70 | 6.08 | 70 | M KOH |
| Fe-SA-NC | 0.98 | 0.86 | 5.44 | 5.2 | 70 | |
| Fe-SA-NSC | 1.00 | 0.88 | 5.75 | 13.60 | 69 | |
| Pt/C | 0.96 | 0.85 | 5.75 | 5.20 | 72 | |
| Fe-SA-NC | 0.89 | 0.72 | 4.17 | 0.31 | 124 | |
| Fe-SA-NSC | 0.90 | 0.79 | 4.76 | 0.41 | 76 | 0.1 |
| Fe-SA-NSFC | 0.93 | 0.82 | 4.87 | 1.25 | 57 | M $HClO_4$ |
| Pt/C | 0.97 | 0.86 | 4.70 | 5.35 | 70 | |

**Supplementary Table 7.** Summary of reported ORR performance of M-SACs in a 0.1 M KOH electrolyte solution.

| Catalyst | Onset potential (V) | $E_{1/2}$ (V) | $J_k$ (mA $cm^{-2}$) | Tafel slope (mV $decade^{-1}$) | Ref. |
|---|---|---|---|---|---|
| Fe-SA-NSFC | 1.01 | 0.91 | 61.5 at 0.85 V | 53 | This work |
| Fe-SA-NSC | 1.01 | 0.88 | 13.6 at 0.85 V | 69 | This work |
| Fe-SA-NC SAC | 0.98 | 0.90 | / | 48 | 18 |
| Co-ISAS/p-CN | 0.90 | 0.838 | 5.2 at 0.83 V | / | 28 |
| Cu-N-C | 0.96 | 0.869 | 11.8 at 0.85 V | / | 29 |
| Fe-N-C-900 | 0.99 | 0.927 | 29.0 at 0.85 V | / | 4 |
| SA-Fe-NHPC | 1.01 | 0.93 | 57.2 at 0.85 V | 57.2 | 15 |
| Fe-SAs/NPS-HC | / | 0.912 | 71.9 at 0.85 V | 36 | 30 |
| $Fe-N_4$ -NC | 0.972 | 0.885 | / | | 31 |
| Fe-ISAs/CN | / | 0.9 | 37.83 at 0.85 V | 58 | 32 |
| Fe@Aza-PON | / | 0.839 | ~ 4.3 at 0.85 V | 60 | 33 |
| $Fe_2$-Z8-C | 0.985 | 0.871 | / | | 34 |
| SA-Fe-HPC | / | 0.89 | 3.72 at 0.9V | 49 | 35 |
| Fe-ISA/SNC | / | 0.896 | 100.7 at 0.85 V | 44.0 | 36 |
| Zn/CoN-C | 1.004V | 0.861V | / | 67.0 | 37 |
| pfSAC-Fe-X | / | 0.910 | 25.86 at 0.85 V | 31.7 | 38 |
| $FeCl_1N_4$/CNS | / | 0.921 | 41.11 at 0.85 V | 51.0 | 14 |
| S,N-Fe/N/C-CNT | 0.85 | | ~ 7.4 at 0.85 V | 82 | 39 |
| Fe@C-FeNCs-2 | / | 0.899 | 41.6 at 0.8 V | 68 | 40 |
| Fe/N/C-SCN | / | 0.836 | ~ 28 at 0.8 V<br>~ 1.9 at 0.85 V | 66 | 41 |
| Co SAs/N-C(900) | / | 0.881 | 21.2 at 0.8 V | 75 | 23 |
| SA-Fe/NG | / | 0.88 | / | 82 | 42 |

**Supplementary Table 8**. Summary of reported ORR performance of M-SACs in acid electrolyte solutions.

| Catalysts | Onset potential (V) | $E_{1/2}$ (V) at 900 rmp | $J_k$ (mA $cm^{-2}$) | Tafel slope (mV $decade^{-1}$) | Electrolyte | Ref. |
|---|---|---|---|---|---|---|
| Fe-SA-NSFC | 0.95 | 0.82 | 1.25 at 0.85 V | 57 | 0.1M $HClO_4$ | This work |
| Fe-SA-NSC | 0.91 | 0.79 | 0.41 at 0.85 V | 76 | 0.1M $HClO_4$ | This work |
| SA-Fe-NHPC | / | 0.76, 1600rmp | / | 48 | 0.1M $HClO_4$ | 15 |
| Fe/SNC | / | 0.77 | / | / | 0.5M $H_2SO_4$ | 43 |
| Fe-SAs/NPSHC | / | 0.80, 1600rmp | 21.9 at 0.75 V | / | 0.5M $H_2SO_4$ | 30 |
| 1.5Fe-ZIF | 0.98 | 0.88 | | | 0.5M $H_2SO_4$ | 44 |
| Co–N–C@F127 | 0.93 | 0.84 | 71.9 at 0.85 V | / | 0.5M $H_2SO_4$ | 16 |
| Fe/N/C/NF | 0.93 | 0.8, 1600rmp | | 67 | 0.5M $H_2SO_4$ | 45 |
| (Fe,Co)/N-C | 1.06 | 0.863,1600rmp | 2.842 at 0.9 V | 66 | 0.1M $HClO_4$ | 46 |
| Co-N-GA | / | 0.73,1600rmp | ~4.39 at 0.75 V | / | 0.5M $H_2SO_4$ | 33 |
| 20Mn-NC-second | / | 0.8 | | / | 0.5M $H_2SO_4$ | 11 |
| SA-Fe-HPC | / | 0.81,1600rmp | | / | 0.1M $H_2SO_4$ | 35 |
| Fe-ZIF derived catalyst (50 nm) | / | 0.85 | ~2.1 at 0.85V | / | 0.5M $H_2SO_4$ | 47 |
| ISAS-Co/HNCS | / | 0.773,1600rpm | 12.8 at 0.75 V | / | 0.5M $H_2SO_4$ | 48 |
| SA-Fe-N nanosheets | 0.941 | 0.812 | / | 62 | 0.5M $H_2SO_4$ | 4 |
| FeSAs/PTF-600 | 0.89 | ~ 0.73,1600rpm | / | / | 0.1M $HClO_4$ | 49 |
| Co-N-C | 0.89 | 0.73,1600rpm | / | / | 0.1M $HClO_4$ | 50 |
| ZIF-NC-0.5Fe-700 | / | 0.84 | / | / | 0.5M $H_2SO_4$ | 51 |
| Fe-N-C-3 | / | 0.805 | | / | | 52 |
| FeNS/HPC | / | 0.778 | | 72 | 0.1M $HClO_4$ | 53 |
| SA-Fe/NG | / | 0.80 | 15.6 at 0.75 V | 77 | 0.1M $HClO_4$ | 42 |
| Fe-N-C-950 | 0.9 | 0.78, 1600rpm | 2.7 at 0.8 V | / | 0.1M $HClO_4$ | 54 |
| Fe-SA-NC-Phen-PANI | / | 0.8 | / | / | | 55 |
| Fe0.5-N/CDC-2 | / | 0.81, 1600rmp | 17.2 at 0.8 V | / | 0.5M $H_2SO_4$ | 56 |
| FeSAs/PTF-400 | 0.89 | 0.75, 1600rmp | / | 81 | | 56 |
| (CM+PANI)-Fe-C | | 0.8 | / | / | 0.5M $H_2SO_4$ | 57 |

**Supplementary Table 9.** Comparison of estimated TOFs of our catalysts with the reported state-of-the-art noble metal-free catalysts.

| Catalyst | Electrolyte | TOF (e $s^{-1}$ $site^{-1}$) | Ref. |
|---|---|---|---|
| Fe-SA-NSFC | 0.1 M KOH | 0.22 at 0.85 V | This work |
| Fe-SA-NSFC | 0.1 M $HClO_4$ | 0.17 at 0.8 V | This work |
| CNT/PC (Fe) | 0.1 M KOH | 0.638 at 0.9 V | 58 |
| Fe-ISAs/CN | 0.1 M KOH | 2.48 at 0.8 V | 32 |
| Fe-N-C-950 | 0.1 M $HClO_4$ | 1.27 at 0.8 V | 54 |
| 0.5Fe-950 | 0.1 M $H_2SO_4$ | 0.33 at 0.8 V | 59 |
| CNT/PC | 0.1 M $HClO_4$ | 0.22 at 0.8 V | 58 |
| LTHT-FeP aerogel | 0.1 M KOH | 0.25 at 0.8 V | 60 |

**Fe-SA-NC structure:** The structure of $FeN_4$ was established for Fe-SA-NC according to the literature[61]. The two-dimensional model of a 4×3 graphene hexagonal supercell, consisting of 39 atoms, was separated by a vacuum region of 15 Å along the direction normal to the sheet plane to avoid artificial interactions between graphene layers[62]. Two neighboring carbon atoms were removed to anchor an iron atom, and four carbon atoms were replaced by N atoms, forming the $FeN_4$ site between two adjacent layers.

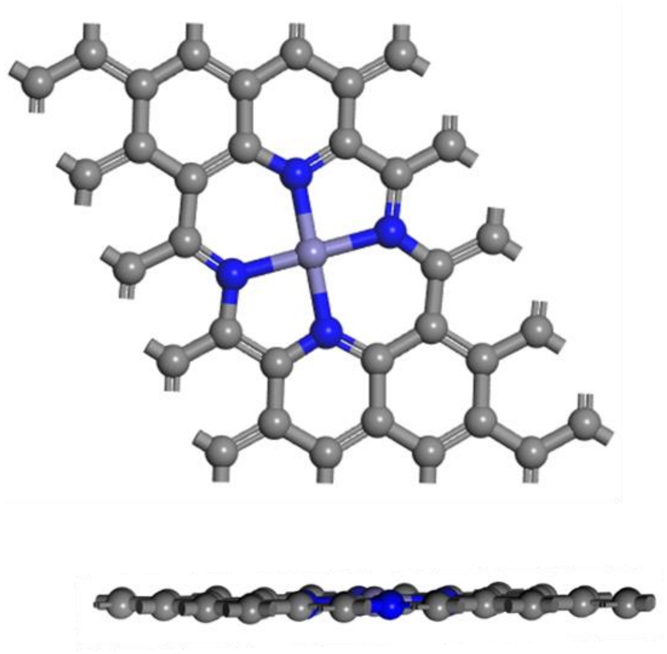

**Supplementary Figure 19**. **Optimized model for Fe-SA-NC.** Here, the gray, purple and blue spheres represent C, Fe and N atoms, respectively.

**Fe-SA-NSC structure:** The Fe-SA-NSC structure was constructed based on the Fe-SA-NC structure. A previous work has proven that the formation of a S-doped configuration on a graphitic $FeN_4$ structure ($FeN_4$-$S_1$) is energetically unfavorable.[63] The incorporation of S-dopant into the graphitic-$FeN_4$ system induces strong geometrical reconstructions that break the planarity of the original structure. In addition, the calculated energy of formation of the $FeN_4$-$S_1$ configuration is positive, indicating that the experimental preparation of the $FeN_4$-$S_1$ structure is challenging.[36, 64] The $FeN_4$ center with two S atom structures are thus usually considered for the S-doped $FeN_4$ (Fe-$N_4$-$S_2$)[30, 36]. In this work, S atoms were built into -C-S-C- bonds (based on the XPS result). Thus, four possible structures were established for the $FeN_4$-$S_2$ configuration and then optimized. Among these arrangements, the $FeN_4$-$S_2$-1 was proposed as a most possible structure for Fe-SA-NSC due to the lowest calculated energy of formation (-4.17 eV).

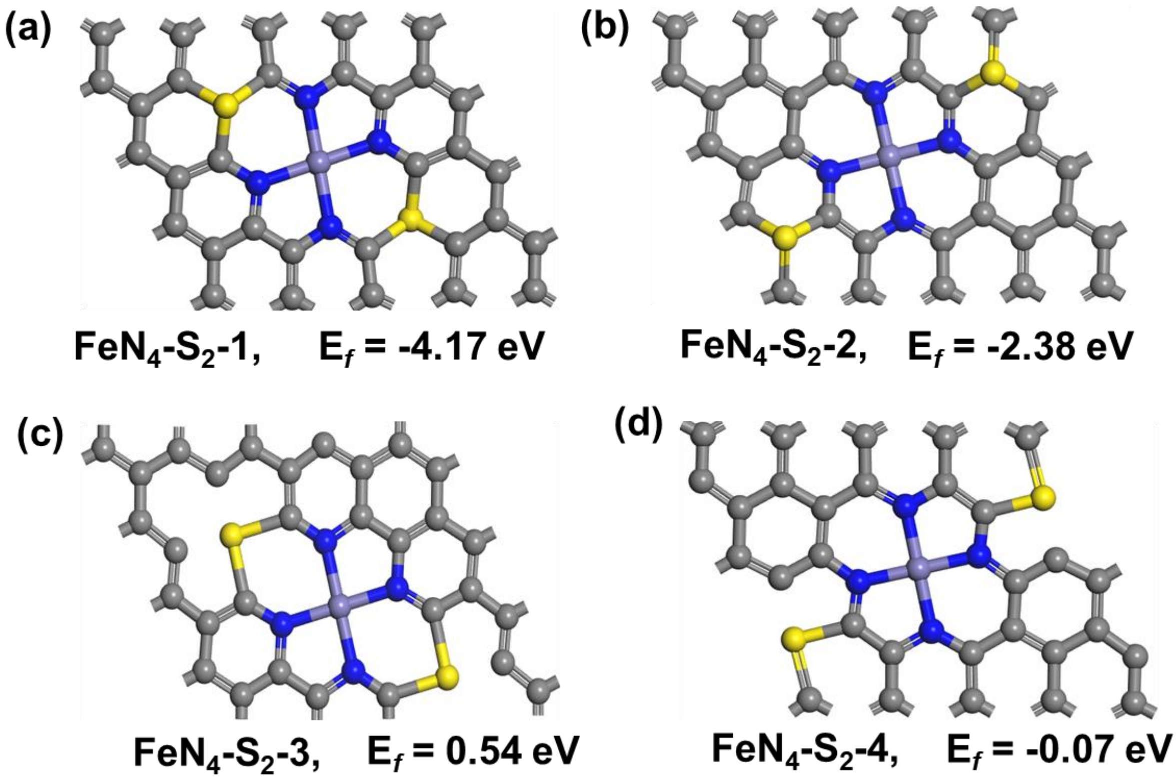


**Supplementary Figure 20**. **Four possible models for Fe-SA-NSC**. **(a)** $FeN_4$-$S_2$-1, **(b)** $FeN_4$-$S_2$-2, **(c)** $FeN_4$-$S_2$-3 and **(d)** $FeN_4$-$S_2$-4. Here, the gray, purple, blue and yellow spheres represent C, Fe N and S atoms, respectively.

**Fe-SA-NSFC structure**: The Fe-SA-NSFC structure was determined based on the Fe-SA-NSC structure. According to the XPS result, the atomic percentages of S and F dopants were almost the same in the Fe-SA-NSFC. Therefore, a $FeN_4$-$S_2$-$F_2$ structure was considered. Two possible arrangements were shown in Supplementary Figure 21. Due to the lower calculated energy of formation -4.52 eV, $FeN_4$-$S_2$-$F_2$-1 was proposed as the more realistic structure for Fe-SA-NSFC.

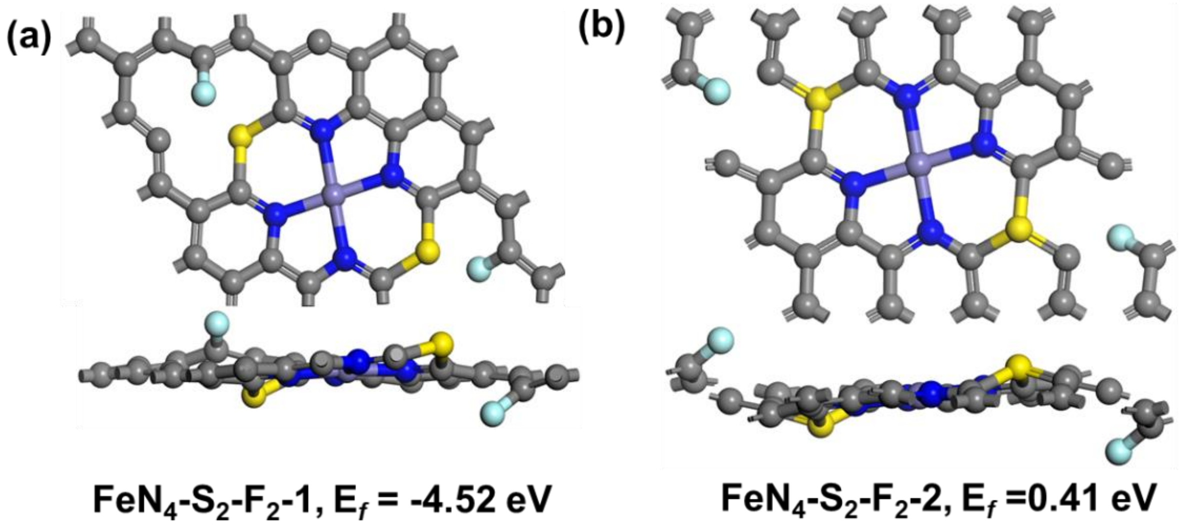


**Supplementary Figure 21**. **Two possible models for Fe-SA-NSFC**. **(a)** $FeN_4$-$S_2$-$F_2$-1 and **(b)** $FeN_4$-$S_2$-$F_2$-2. Here the gray, purple, blue, yellow and light blue spheres represent C, Fe, N, S and F atoms, respectively.

The structures for Fe-SA-NC, Fe-SA-NSC and Fe-SA-NSFC were thus determined and shown in **Supplementary Figure 22.**

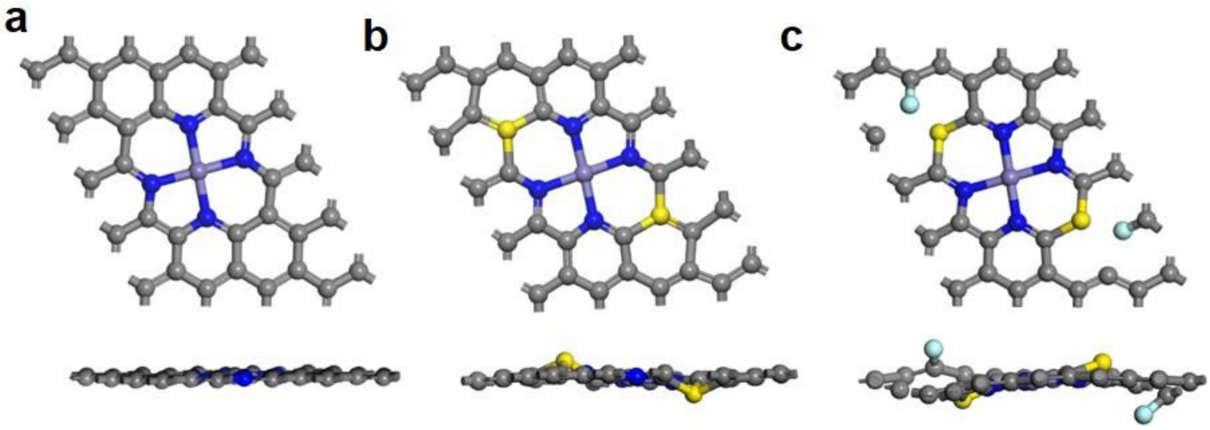


**Supplementary Figure 22**. **Optimized models of catalysts**. **(a)** Fe-SA-NC, **(b)** Fe-SA-NSC and **(c)** Fe-SA-NSFC used for DFT calculations. Here, the gray, purple, blue, yellow and light blue spheres represent C, Fe, N, S and F atoms, respectively. The model of Fe-SA-NSFC showed a higher degree of distortion in the carbon planes compared with those of Fe-SA-NSC and Fe-SA-NC.

**Supplementary Table 10.** The summary of Mulliken charges and OH***** adsorption energies in Fe-SA-NC, Fe-SA-NSC and Fe-SA-NSFC.

| Structure | Mulliken charge | OH* adsorption energy (eV) |
|---|---|---|
| Fe-SA-NC | 1.13 | -0.31 |
| Fe-SA-NSC | 1.15 | -0.26 |
| Fe-SA-NSFC | 1.16 | +0.52 |

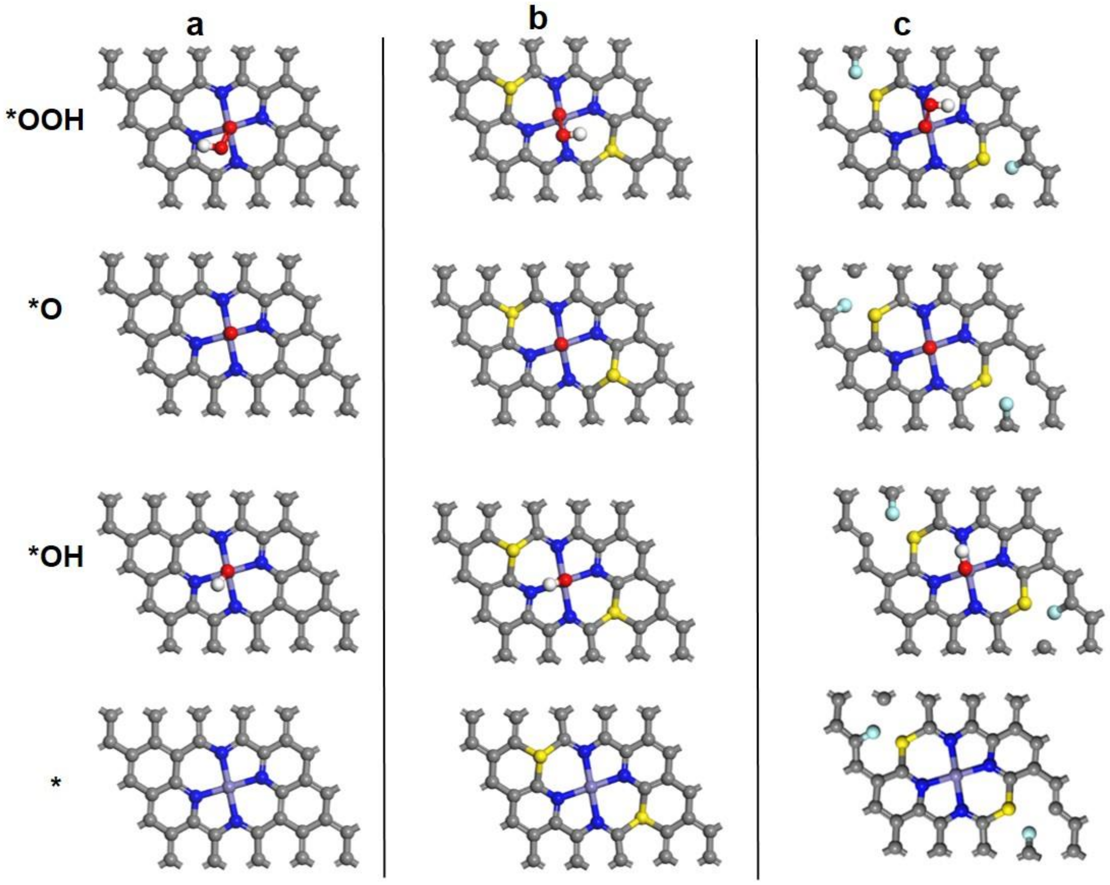


**Supplementary Figure 23. Adsorption structures of ORR intermediates** on **(a)** Fe-SA-NC, **(b)** Fe-SA-NSC and **(c)** Fe-SA-NSFC.

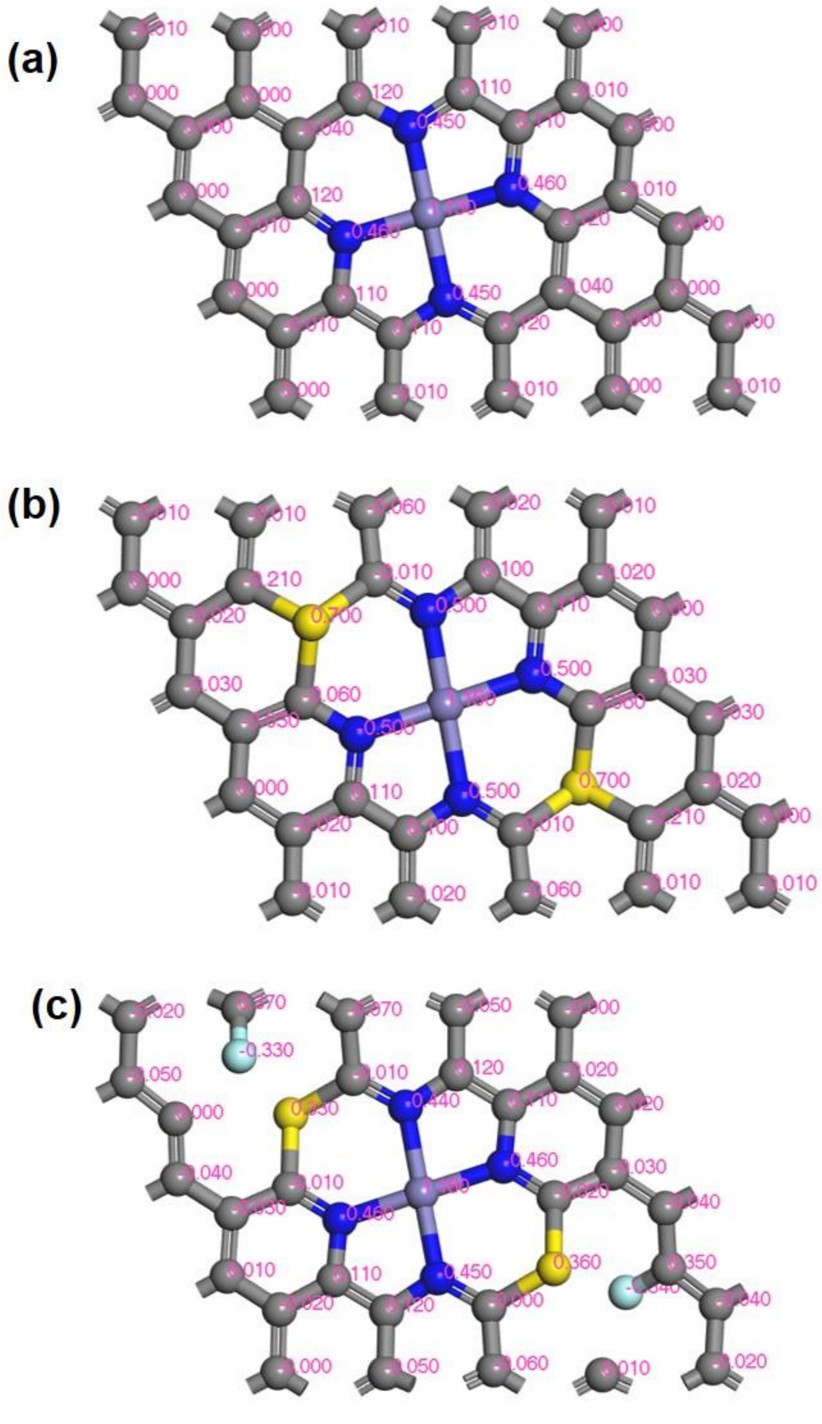


**Supplementary Figure 24.** Mulliken charge distributions of **(a)** Fe-SA-NC, **(b)** Fe-SA-NSC and **(c)** Fe-SA-NSFC.

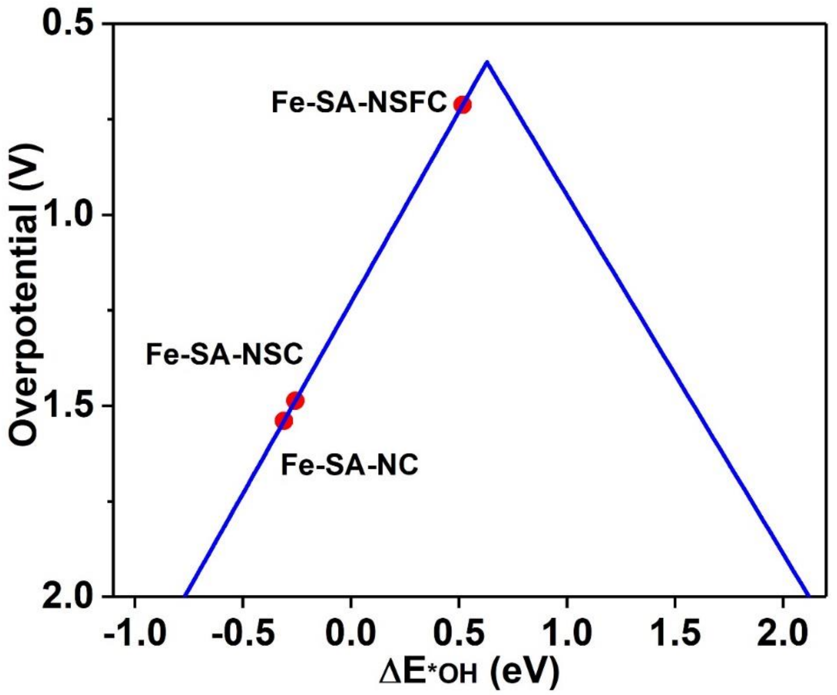


**Supplementary Figure 25**. Volcano plots of the overpotential as a function of the $\Delta E_{*OH}$ associated with Fe-SA-NC, Fe-SA-NSC and Fe-SA-NSFC.

**Supplementary Table 11**. Summary of reported performance of Zn-air batteries using various electrocatalysts.

| Catalyst | Open circuit voltage (V) | Peak power density (mW $cm^{-2}$) | Durability (h) | Reference |
|---|---|---|---|---|
| Fe-SA-NSFC | 1.48 | 247.7 | 240 | This work |
| Fe-SAs/NPS-HC | 1.45 | 195.0 | ~55.6 | 30 |
| SA-Fe-NHPC | 1.52 | 266.4 | 240 | 15 |
| SA-Fe/NG | / | 91 | 20 | 42 |
| meso/micro-FeCo-Nx-CN | / | 150 | 44 | 65 |
| Co-Nx-C | / | 152 | >60 | 66 |
| Mn/Fe-HIB-MOF | / | 195 | 1000 | 67 |
| $Fe-N_4$ SAs/NPC | 1.45 | 232 | 36 | 31 |
| NCo@CNT-NF700 | / | 220 | 133 | 68 |
| (Fe, Co)/CNT | / | 260 | / | 69 |
| CoNi-SAs/NC | / | 101.4 | >30 | 70 |
| Co/Co–N–C | 1.41 | 132 | ~330 | 71 |

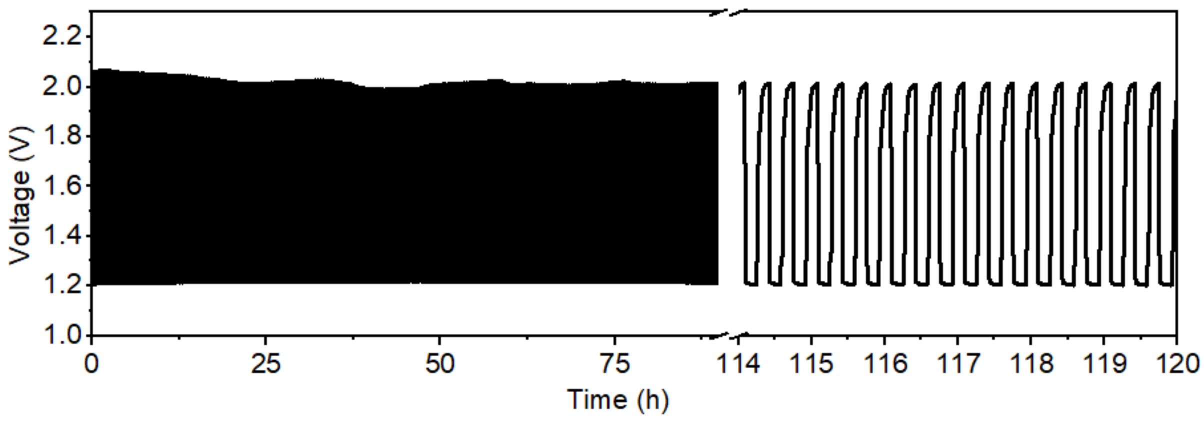


**Supplementary Figure 26.** Charge–discharge cycling performance of the rechargeable Zn-air battery using Fe-SA-NSFC in air electrode at a constant charge–discharge current density of 10 mA cm$^{-2}$**.**

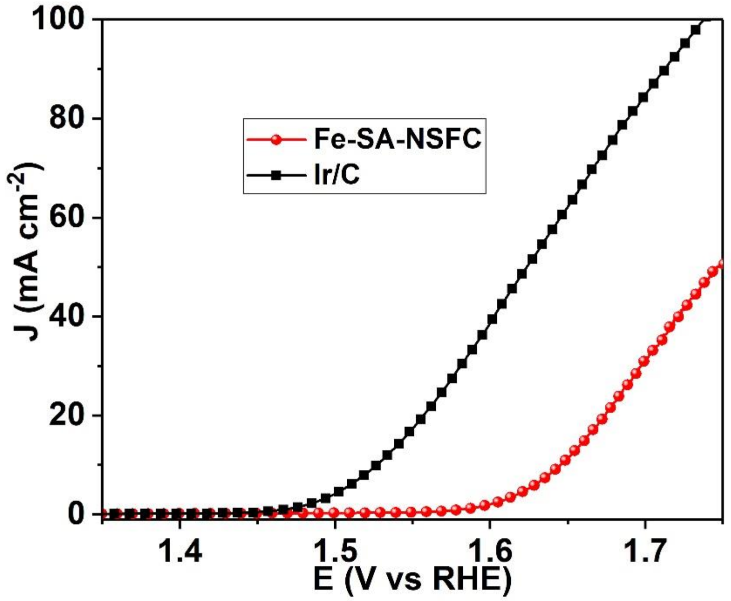


**Supplementary Figure 27**. **I-R (current-resistance)-corrected oxygen evolution reaction (OER) polarization curves of Fe-SA-NSFC and Ir/C (Ir: 20 wt%).** They were collected from 1.0 to 2.0 V (vs RHE) with a scan rate of 2 mV s$^{-1}$ and an electrode rotation speed of 1600 rpm at in a 1.0 M KOH electrolyte solution. The overpotential of Fe-SA-NSFC was 430 mV at a current density of 10 mA cm$^{-2}$, which was significantly higher than that of commercial Ir/C catalyst (304 mV).

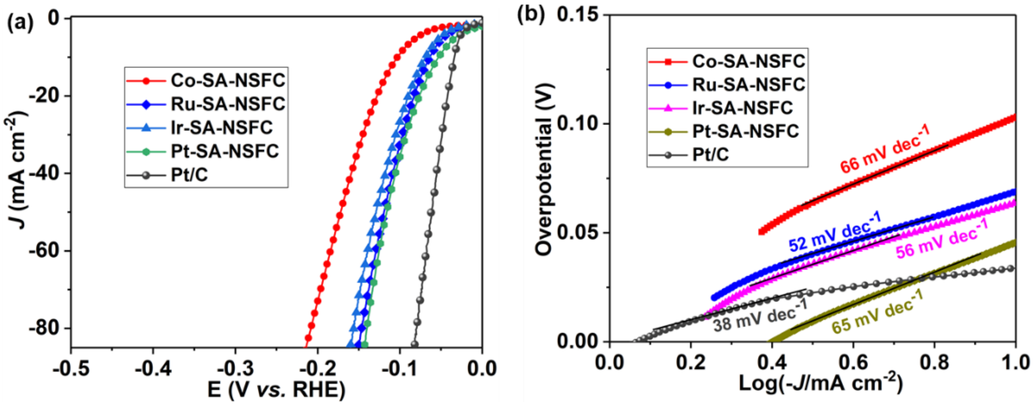


**Supplementary Figure 28. Electrochemical HER performance of catalysts in a 0.5 M $H_2SO_4$ electrolyte solution.** (a) IR-corrected HER polarization curves and (b) corresponding Tafel plots of Co-SA-NSFC, Ru-SA-NSFC, Ir-SA-NSFC, Pt-SA-NSFC and commercial Pt/C catalysts.

**Supplementary Table 12**. Comparison of HER performance of our catalysts with the reported state-of-the-art catalysts.

| Catalyst | $\eta_0$ (mV) | $\eta_j$ (mV) at 10 mA cm$^{-2}$ | Tafel slope (mV decade$^{-1}$) | Electrolyte | Ref. |
|---|---|---|---|---|---|
| Co-SA-NSFC | 29 | 104 | 66 | 0.5M $H_2SO_4$ | This work |
| Ru-SA-NSFC | 18 | 62 | 52 | 0.5M $H_2SO_4$ | This work |
| Ir-SA-NSFC | 18 | 67 | 56 | 0.5M $H_2SO_4$ | This work |
| Pt-SA-NSFC | 10 | 45 | 65 | 0.5M $H_2SO_4$ | This work |
| Pt/C (20 $wt_{Pt}$%) | 7 | 33 | 38 | 0.5M $H_2SO_4$ | |
| Co@NG-800 | | 286 | 118 | 0.5M $H_2SO_4$ | 72 |
| CoFe-CDs | / | 334 | 218.4 | 1 M KOH | 73 |
| CoNx/C | | 133 | ~57 | 0.5M $H_2SO_4$ | 74 |
| $Co_1$/PCN | 38 | 154 | 52 | 0.5M $H_2SO_4$ | 75 |
| Co-SA/P-in situ | / | 98 | 47 | 0.5M $H_2SO_4$ | 76 |
| CoP/NCNHP | / | 140 | 53 | 0.5M $H_2SO_4$ | 77 |
| [Ru(SA)+Ru(NP)@$RuN_x$@$G_N$]/ $G_N$ | 0 | 31 | | 0.1M $HClO_4$ | 78 |
| RuPP@fCF | / | 180 | 158 | 1 M $H_2SO_4$ | 79 |
| Ru@CF | / | 99 | 70 | 1 M KOH | 80 |
| Ru-CoCH@CF | / | 66 | 65 | 1 M KOH | 80 |
| Ru@C | / | 86 | 95.7 | 1 M KOH | 73 |
| Ir@G-750 | / | 28 | 29 | 0.5M $H_2SO_4$ | 81 |
| Ir/N-G-750 | / | 19 | 26 | 0.5M $H_2SO_4$ | 81 |
| IrHNC | / | 4.5 | | 0.5M $H_2SO_4$ | 82 |
| C-$Ir_1$/$Co_{0.8}Fe_{0.2}Se_2$ | / | 8 | | 1 M KOH | 83 |
| $Pt_1$/OLC | / | ~38 | 36 | 0.5M $H_2SO_4$ | 84 |
| Pt/np-$Co_{0.85}$Se | / | 55 | 35 | PBS, pH=7.0 | 85 |
| $Pt_1$/hNCNC | / | 15 | 24 | 0.5M $H_2SO_4$ | 86 |
| Pt/$Ti_3C_3T_x$-700 | / | 55.8 | 43.9 | 0.1M $HClO_4$ | 87 |
| PtNP/OMC | / | $(70)_{100}$ | 32 | 0.5M $H_2SO_4$ | 88 |
| Pt13Cu73Ni14/CNF@CF | / | $(67)_5$ | 38 | 0.5M $H_2SO_4$ | 89 |

$\eta$0: Onset potential. $\eta_j$: Overpotential at a current density of j.

**Supplementary Table 13.** Cartesian coordinates of Fe-$N_4$ for Fe-SA-NC. Space group: *P*1; a = 10.0 Å, b = 12.4 Å, c = 16.6 Å, α = 90.2°, β = 88.5° and γ = 117.3°.

| Atom | *x/a* | *y/b* | *z/c* |
|---|---|---|---|
| C | 2.1895 | 3.1373 | 1.7813 |
| C | 2.1822 | 3.3334 | 1.7818 |
| C | 2.2675 | 3.2664 | 1.7815 |
| C | 2.1985 | 2.9403 | 1.7809 |
| C | 2.2827 | 2.8721 | 1.7809 |
| C | 2.4467 | 2.9404 | 1.7809 |
| C | 2.5325 | 2.8732 | 1.7809 |
| C | 2.6967 | 2.9433 | 1.7813 |
| C | 2.7844 | 2.8783 | 1.7812 |
| C | 2.9485 | 2.9425 | 1.7814 |
| C | 3.0340 | 2.8765 | 1.7812 |
| C | 2.4415 | 3.1424 | 1.7812 |
| N | 2.4262 | 3.3355 | 1.7815 |
| C | 2.5170 | 3.2768 | 1.7817 |
| C | 2.2772 | 3.0722 | 1.7810 |
| C | 2.6912 | 3.1434 | 1.7818 |
| C | 2.6865 | 3.3474 | 1.7821 |
| C | 2.5272 | 3.0752 | 1.7813 |
| C | 2.9398 | 3.1391 | 1.7818 |
| C | 2.9377 | 3.3405 | 1.7821 |
| C | 3.0197 | 3.2706 | 1.7820 |
| C | 2.7754 | 3.0753 | 1.7817 |
| C | 3.0254 | 3.0731 | 1.7815 |
| C | 2.1961 | 3.5326 | 1.7817 |
| C | 2.2873 | 3.6681 | 1.7812 |
| C | 2.4569 | 3.7388 | 1.7809 |
| N | 2.5477 | 3.6801 | 1.7807 |
| N | 2.2693 | 3.4601 | 1.7817 |
| N | 2.7045 | 3.5554 | 1.7818 |
| C | 2.7064 | 3.7492 | 1.7809 |
| C | 2.7917 | 3.6821 | 1.7813 |
| C | 2.9464 | 3.5433 | 1.7818 |
| C | 2.9542 | 3.7450 | 1.7813 |
| C | 3.0361 | 3.6750 | 1.7814 |
| C | 2.7777 | 3.4829 | 1.7821 |
| C | 3.0274 | 3.4723 | 1.7819 |
| Fe | 2.4868 | 3.5077 | 1.7841 |
| C | 0.7711 | 0.2778 | 0.7821 |
| C | 0.2027 | 0.7377 | 0.7811 |

**Supplementary Table 14.** Cartesian coordinates of Fe-$N_4$-$S_2$-1 for Fe-SA-NSC. Space group: *P*1; a = 10.0 Å, b = 12.4 Å, c = 16.6 Å, α = 90.2˚, β = 88.5˚, and γ = 117.3˚.

| Atom | *x/a* | *y/b* | *z/c* |
|---|---|---|---|
| C | 2.2094 | 3.1437 | 1.7791 |
| C | 2.2004 | 3.3367 | 1.7797 |
| C | 2.2843 | 3.2717 | 1.7771 |
| C | 2.2220 | 2.9508 | 1.7829 |
| C | 2.3081 | 2.8852 | 1.7870 |
| C | 2.4720 | 2.9516 | 1.7842 |
| C | 2.5614 | 2.8868 | 1.7839 |
| C | 2.7238 | 2.9546 | 1.7826 |
| C | 2.8120 | 2.8906 | 1.7842 |
| C | 2.9731 | 2.9526 | 1.7826 |
| C | 3.0593 | 2.8875 | 1.7833 |
| C | 2.4600 | 3.1475 | 1.7794 |
| N | 2.4400 | 3.3396 | 1.7722 |
| C | 2.5212 | 3.2760 | 1.7739 |
| C | 2.2976 | 3.0797 | 1.7807 |
| C | 2.7133 | 3.1491 | 1.7762 |
| S | 2.7085 | 3.3607 | 1.7329 |
| C | 2.5494 | 3.0827 | 1.7792 |
| C | 2.9620 | 3.1468 | 1.7799 |
| C | 2.9585 | 3.3454 | 1.7756 |
| C | 3.0403 | 3.2763 | 1.7795 |
| C | 2.7994 | 3.0835 | 1.7803 |
| C | 3.0483 | 3.0817 | 1.7804 |
| C | 2.2048 | 3.5247 | 1.7843 |
| S | 2.3129 | 3.6737 | 1.8304 |
| C | 2.5001 | 3.7582 | 1.7893 |
| N | 2.5814 | 3.6947 | 1.7915 |
| N | 2.2851 | 3.4618 | 1.7835 |
| N | 2.7362 | 3.5725 | 1.7807 |
| C | 2.7372 | 3.7626 | 1.7866 |
| C | 2.8210 | 3.6975 | 1.7842 |
| C | 2.9744 | 3.5564 | 1.7842 |
| C | 2.9811 | 3.7579 | 1.7841 |
| C | 3.0629 | 3.6889 | 1.7877 |
| C | 2.8164 | 3.5095 | 1.7795 |
| C | 3.0470 | 3.4780 | 1.7794 |
| Fe | 2.5106 | 3.5172 | 1.7822 |
| C | 0.7996 | 0.2789 | 0.7703 |
| C | 0.2218 | 0.7553 | 0.7928 |

**Supplementary Table 15.** Cartesian coordinates of Fe-$N_4$-$S_2$-2 for Fe-SA-NSC. Space group: *P*1; a = 10.0 Å, b = 12.4 Å, c = 16.6 Å, α = 90.2˚, β = 88.5˚, and γ = 117.3˚.

| Atom | *x/a* | *y/b* | *z/c* |
|---|---|---|---|
| S | 2.2008 | 3.1424 | 1.7243 |
| C | 2.1847 | 3.3368 | 1.7813 |
| C | 2.2794 | 3.2801 | 1.7755 |
| C | 2.2006 | 2.9390 | 1.7835 |
| C | 2.2817 | 2.8706 | 1.7925 |
| C | 2.4448 | 2.9401 | 1.7936 |
| C | 2.5289 | 2.8728 | 1.7864 |
| C | 2.6877 | 2.9474 | 1.7771 |
| S | 2.7789 | 2.8747 | 1.7244 |
| C | 2.9589 | 2.9448 | 1.7674 |
| C | 3.0372 | 2.8754 | 1.7750 |
| C | 2.4444 | 3.1427 | 1.7865 |
| N | 2.4321 | 3.3380 | 1.7865 |
| C | 2.5217 | 3.2781 | 1.7863 |
| C | 2.2864 | 3.0684 | 1.7773 |
| C | 2.6910 | 3.1448 | 1.7920 |
| C | 2.6881 | 3.3472 | 1.7897 |
| C | 2.5278 | 3.0752 | 1.7934 |
| C | 2.9374 | 3.1403 | 1.7752 |
| C | 2.9384 | 3.3406 | 1.7871 |
| C | 3.0192 | 3.2712 | 1.7798 |
| C | 2.7731 | 3.0766 | 1.7834 |
| C | 3.0163 | 3.0710 | 1.7674 |
| C | 2.1943 | 3.5338 | 1.7896 |
| C | 2.2850 | 3.6681 | 1.7898 |
| C | 2.4517 | 3.7374 | 1.7861 |
| N | 2.5412 | 3.6774 | 1.7866 |
| N | 2.2670 | 3.4622 | 1.7878 |
| N | 2.7064 | 3.5531 | 1.7883 |
| C | 2.6950 | 3.7355 | 1.7758 |
| C | 2.7893 | 3.6786 | 1.7812 |
| C | 2.9464 | 3.5426 | 1.7893 |
| C | 2.9549 | 3.7444 | 1.7795 |
| C | 3.0350 | 3.6748 | 1.7870 |
| C | 2.7789 | 3.4815 | 1.7898 |
| C | 3.0269 | 3.4727 | 1.7894 |
| Fe | 2.4863 | 3.5075 | 1.7922 |
| C | 0.7721 | 0.2780 | 0.7903 |
| C | 0.2008 | 0.7373 | 0.7907 |

**Supplementary Table 16.** Cartesian coordinates of Fe-$N_4$-$S_2$-3 for Fe-SA-NSC. Space group: *P*1; a = 10 Å, b = 12.4 Å, c = 16.6 Å, α = 90.2°, β = 88.5°, and γ = 117.3°.

| Atom | *x/a* | *y/b* | *z/c* |
|---|---|---|---|
| C | 2.0726 | 3.1421 | 1.7835 |
| C | 2.0675 | 3.3421 | 1.7831 |
| C | 2.1520 | 3.2723 | 1.7834 |
| C | 2.0855 | 2.9408 | 1.7835 |
| C | 2.1835 | 2.8819 | 1.7836 |
| C | 2.3389 | 2.9496 | 1.7829 |
| C | 2.4315 | 2.8876 | 1.7824 |
| C | 2.5918 | 2.9563 | 1.7824 |
| C | 2.6754 | 2.8864 | 1.7826 |
| C | 2.8380 | 2.9483 | 1.7832 |
| C | 2.9226 | 2.8800 | 1.7834 |
| C | 2.3166 | 3.1410 | 1.7829 |
| N | 2.3126 | 3.3377 | 1.7829 |
| C | 2.3875 | 3.2679 | 1.7825 |
| C | 2.1563 | 3.0723 | 1.7832 |
| C | 2.5646 | 3.1468 | 1.7836 |
| S | 2.6144 | 3.3146 | 1.7874 |
| C | 2.4093 | 3.0791 | 1.7830 |
| C | 2.8255 | 3.1486 | 1.7837 |
| C | 2.8254 | 3.3536 | 1.7834 |
| C | 2.9033 | 3.2799 | 1.7834 |
| C | 2.6625 | 3.0878 | 1.7831 |
| C | 2.9100 | 3.0802 | 1.7836 |
| C | 2.0715 | 3.5339 | 1.7819 |
| S | 2.1335 | 3.7140 | 1.7863 |
| C | 2.3605 | 3.7607 | 1.7822 |
| N | 2.4354 | 3.6908 | 1.7825 |
| N | 2.1537 | 3.4707 | 1.7825 |
| N | 2.5946 | 3.5580 | 1.7820 |
| C | 2.5961 | 3.7563 | 1.7825 |
| C | 2.6806 | 3.6865 | 1.7823 |
| C | 2.8348 | 3.5510 | 1.7820 |
| C | 2.8448 | 3.7488 | 1.7828 |
| C | 2.9228 | 3.6751 | 1.7830 |
| C | 2.6768 | 3.4948 | 1.7819 |
| C | 2.9136 | 3.4778 | 1.7821 |
| Fe | 2.3741 | 3.5144 | 1.7808 |

**Supplementary Table 17.** Cartesian coordinates of Fe-$N_4$-$S_2$-4 for Fe-SA-NSC. Space group: *P*1; a = 10.0 Å, b = 12.4 Å, c = 16.6 Å, α = 90.2°, β = 88.5°, and γ = 117.3°.

| Atom | *x/a* | *y/b* | *z/c* |
|---|---|---|---|
| S | 2.2401 | 3.1064 | 1.7729 |
| C | 2.1935 | 3.3333 | 1.7806 |
| C | 2.2906 | 3.2724 | 1.7797 |
| C | 2.1798 | 2.9346 | 1.7806 |
| C | 2.2752 | 2.8741 | 1.7806 |
| C | 2.4443 | 2.9397 | 1.7813 |
| C | 2.5177 | 2.8619 | 1.7830 |
| S | 2.7338 | 2.9092 | 1.7895 |
| C | 2.9430 | 2.9476 | 1.7829 |
| C | 3.0200 | 2.8720 | 1.7828 |
| C | 2.4563 | 3.1536 | 1.7800 |
| N | 2.4411 | 3.3409 | 1.7820 |
| C | 2.5337 | 3.2822 | 1.7814 |
| C | 2.6987 | 3.1414 | 1.7822 |
| C | 2.6979 | 3.3501 | 1.7820 |
| C | 2.5296 | 3.0758 | 1.7815 |
| C | 2.9539 | 3.1436 | 1.7797 |
| C | 2.9450 | 3.3400 | 1.7812 |
| C | 3.0310 | 3.2728 | 1.7799 |
| C | 2.7941 | 3.0810 | 1.7819 |
| C | 3.0309 | 3.0680 | 1.7794 |
| C | 2.1929 | 3.5312 | 1.7815 |
| C | 2.2759 | 3.6654 | 1.7814 |
| C | 2.4401 | 3.7334 | 1.7819 |
| N | 2.5327 | 3.6747 | 1.7817 |
| N | 2.2701 | 3.4602 | 1.7818 |
| N | 2.7038 | 3.5553 | 1.7819 |
| C | 2.6833 | 3.7431 | 1.7835 |
| C | 2.7804 | 3.6822 | 1.7826 |
| C | 2.9463 | 3.5435 | 1.7819 |
| C | 2.9429 | 3.7428 | 1.7830 |
| C | 3.0288 | 3.6755 | 1.7820 |
| C | 2.7809 | 3.4843 | 1.7820 |
| C | 3.0275 | 3.4721 | 1.7815 |
| Fe | 2.4870 | 3.5078 | 1.7816 |
| C | 0.7797 | 0.2772 | 0.7819 |
| C | 0.1941 | 0.7384 | 0.7812 |

**Supplementary Table 18.** Cartesian coordinates of Fe-$N_4$-$S_2$-$F_2$-1 for Fe-SA-NSFC. Space group: *P*1; a = 10.0 Å, b = 12.4 Å, c = 16.6 Å, α = 90.2°, β = 88.5°, and γ = 117.3°.

| Atom | *x/a* | *y/b* | *z/c* |
|---|---|---|---|
| C | 2.0722 | 3.1433 | 1.7861 |
| C | 2.0677 | 3.3383 | 1.7762 |
| C | 2.1490 | 3.2716 | 1.7851 |
| C | 2.0841 | 2.9500 | 1.8020 |
| C | 2.1793 | 2.8974 | 1.8220 |
| C | 2.3336 | 2.9483 | 1.8010 |
| C | 2.4242 | 2.8832 | 1.7928 |
| C | 2.5870 | 2.9511 | 1.7877 |
| C | 2.6744 | 2.8869 | 1.7866 |
| C | 2.8367 | 2.9498 | 1.7886 |
| C | 2.9222 | 2.8838 | 1.7945 |
| C | 2.3203 | 3.1470 | 1.7949 |
| N | 2.3046 | 3.3399 | 1.7916 |
| C | 2.3847 | 3.2763 | 1.7984 |
| C | 2.1589 | 3.0792 | 1.7927 |
| C | 2.5643 | 3.1338 | 1.7853 |
| S | 2.5751 | 3.3499 | 1.8260 |
| F | 2.7261 | 3.3105 | 1.6932 |
| C | 2.4094 | 3.0805 | 1.7917 |
| C | 2.8254 | 3.1459 | 1.7738 |
| C | 2.8493 | 3.3544 | 1.7390 |
| C | 2.9090 | 3.2767 | 1.7629 |
| C | 2.6619 | 3.0815 | 1.7817 |
| C | 2.9102 | 3.0802 | 1.7833 |
| C | 2.0797 | 3.5310 | 1.7645 |
| F | 1.1170 | 3.7959 | 1.8691 |
| S | 2.1782 | 3.6862 | 1.7405 |
| C | 2.3611 | 3.7543 | 1.7809 |
| N | 2.4422 | 3.6911 | 1.7825 |
| N | 2.1544 | 3.4635 | 1.7720 |
| N | 2.5949 | 3.5675 | 1.7864 |
| C | 2.5975 | 3.7588 | 1.7870 |
| C | 2.6802 | 3.6923 | 1.7862 |
| C | 2.8308 | 3.5488 | 1.7680 |
| C | 2.8420 | 3.7526 | 1.7861 |
| C | 2.9043 | 3.6744 | 1.7710 |
| C | 2.6683 | 3.4984 | 1.7852 |
| C | 2.9173 | 3.4793 | 1.7573 |
| Fe | 2.3737 | 3.5151 | 1.7835 |

**Supplementary Table 19.** Cartesian coordinates of Fe-$N_4$-$S_2$-$F_2$-2 for Fe-SA-NSFC. Space group: *P*1; a = 10.0 Å, b = 12.4 Å, c = 16.6 Å, α = 90.2˚, β = 88.5˚, and γ = 117.3˚.

| Atom | *x/a* | *y/b* | *z/c* |
|---|---|---|---|
| C | 2.2075 | 3.1444 | 1.7722 |
| C | 2.2041 | 3.3378 | 1.7881 |
| C | 2.2872 | 3.2721 | 1.7789 |
| C | 2.2062 | 2.9548 | 1.7363 |
| F | 2.2703 | 2.9027 | 1.6882 |
| C | 2.5063 | 2.9673 | 1.7917 |
| C | 2.5691 | 2.8894 | 1.8060 |
| C | 2.7328 | 2.9547 | 1.8111 |
| C | 2.8139 | 2.8900 | 1.7914 |
| C | 2.9733 | 2.9527 | 1.7804 |
| C | 3.0536 | 2.8860 | 1.7613 |
| C | 2.4524 | 3.1450 | 1.7576 |
| N | 2.4435 | 3.3390 | 1.7691 |
| C | 2.5211 | 3.2735 | 1.7611 |
| C | 2.2886 | 3.0795 | 1.7525 |
| F | 2.7511 | 3.1314 | 1.8758 |
| S | 2.7223 | 3.3595 | 1.7279 |
| C | 2.5150 | 3.0670 | 1.7722 |
| C | 2.9679 | 3.1483 | 1.8026 |
| C | 2.9633 | 3.3468 | 1.7758 |
| C | 3.0449 | 3.2782 | 1.7934 |
| C | 2.8152 | 3.0794 | 1.8276 |
| C | 3.0482 | 3.0816 | 1.7835 |
| C | 2.2083 | 3.5267 | 1.7917 |
| S | 2.2992 | 3.6748 | 1.8355 |
| C | 2.5002 | 3.7609 | 1.8018 |
| N | 2.5778 | 3.6954 | 1.7932 |
| N | 2.2894 | 3.4634 | 1.7880 |
| N | 2.7320 | 3.5709 | 1.7751 |
| C | 2.7342 | 3.7623 | 1.7841 |
| C | 2.8173 | 3.6965 | 1.7751 |
| C | 2.9713 | 3.5547 | 1.7812 |
| C | 2.9765 | 3.7561 | 1.7703 |
| C | 3.0580 | 3.6874 | 1.7880 |
| C | 2.8131 | 3.5076 | 1.7716 |
| C | 3.0500 | 3.4795 | 1.7823 |
| Fe | 2.5107 | 3.5172 | 1.7812 |
| C | 0.8157 | 0.2818 | 0.7512 |
| C | 0.2054 | 0.7523 | 0.8127 |

**Supplementary Reference**


1. Bard, A. J. & Faulkner, L. R., *Electrochemical Methods: Fundamentals and Applications* , Wiley, New York, 2001.
2. Silva, T.C.D.E. et al. Polymer-derived Co/Ni-SiOC(N) ceramic electrocatalysts for oxygen reduction reaction in fuel cells. *Catal. Sci. Technol.* **9**, 854-866 (2019).
3. Schonvogel, D. et al. Durability of Electrocatalysts for ORR: Pt on nanocomposite of reduced graphene oxide with FTO versus Pt/C. *J. Electrochem. Soc.* **165**, F3373-F3382 (2018).
4. Miao, Z. P. et al. Atomically Dispersed Fe-$N_x$/C Electrocatalyst Boosts Oxygen Catalysis via a New Metal-Organic Polymer Supramolecule Strategy. *Adv Energy Mater* **8**, 1801226 (2018).
5. http://www.dftb.org
6. Elstner, M. et al. Self-consistent-charge density-functional tight-binding method for simulations of complex materials properties. *Phys. Rev. B* **58**, 7260-7268 (1998).
7. Koskinen, P. & Makinen, V. Density-functional tight-binding for beginners. *Comp. Mater. Sci.* **47**, 237-253 (2009).
8. Tang, W., Sanville, E. & Henkelman, G. A grid-based bader analysis algorithm without lattice bias. *J Phys-Condens Mat.* **21**, 084204 (2009).
9. Norskov, J. K. et al. Origin of the overpotential for oxygen reduction at a fuel-cell cathode. *J. Phys. Chem. B* **108**, 17886-17892 (2004).
10. Wang, L. et al. A sulfur-tethering synthesis strategy toward high-loading atomically dispersed noble metal catalysts. *Sci. Adv.* **5**, eaax6322 (2019).
11. Li, J. Z. et al. Atomically dispersed manganese catalysts for oxygen reduction in proton-exchange membrane fuel cells. *Nat. Catal.* **1**, 935-945 (2018).
12. Ding, Y. X. et al. Cobalt-bridged ionic liquid polymer on a carbon nanotube for enhanced oxygen evolution reaction activity. *Angew. Chem. Int. Ed.* **57**, 3514-3518 (2018).
13. Deng, D. H. et al. A single iron site confined in a graphene matrix for the catalytic oxidation of benzene at room temperature. *Sci. Adv.* **1**, e1500462 (2015).
14. Han, Y. H. et al. Electronic structure engineering to boost oxygen reduction activity by controlling the coordination of the central metal. *Energ. Environ. Sci.***11**, 2348-2352 (2018).
15. Chen, G. B. et al. Zinc-mediated template synthesis of Fe-N-C electrocatalysts with densely accessible Fe-$N_x$ active sites for efficient oxygen reduction. *Adv. Mate.* **32**, 1907399 (2020).
16. He, Y. H. et al. Highly active atomically dispersed $CoN_4$ fuel cell cathode catalysts derived from surfactant-assisted MOFs: carbon-shell confinement strategy. *Energ. Environ. Sci.* **12**, 250-260 (2019).
17. Liu, Y. W. et al. A general strategy for fabricating isolated single metal atomic site catalysts in Y zeolite. *J. Am. Chem. Soc.* **141**, 9305-9311 (2019).
18. Zhao, L. et al. Cascade anchoring strategy for general mass production of high-loading single-atomic metal-nitrogen catalysts. *Nat. Commun.* **10**, 1278 (2019).
19. Choi, C. H. et al. Tuning selectivity of electrochemical reactions by atomically dispersed platinum catalyst. *Nat. Commun.* **7**, 10922 (2016).
20. Zhang, L. H., Han, L. L., Liu, H. X., Liu, X. J. & Luo, J. Potential-cycling synthesis of single platinum atoms for efficient hydrogen evolution in neutral media. *Angew. Chem. Int. Ed.* **56**, 13694-13698 (2017).
21. Li, H. L. et al. Synergetic interaction between neighbouring platinum monomers in $CO_2$ hydrogenation. *Nat. Nanotechnol.* **13**, 411 (2018).

22. Chen, W. L. et al. Neighboring Pt atom sites in an ultrathin FePt nanosheet for the efficient and highly CO-tolerant oxygen reduction reaction. *Nano. Lett.* **18**, 5905-5912 (2018).
23. Yin, P. Q. et al. Single cobalt atoms with precise N-coordination as superior oxygen reduction reaction catalysts. *Angew. Chem. Int. Ed.* **55**, 10800-10805 (2016).
24. Lu, Y. B. et al. Identification of the active complex for CO oxidation over single-atom Ir-on-$MgAl_2O_4$ catalysts. *Nat. Catal.* **2**, 149-156 (2019).
25. Mun, Y. et al. Versatile strategy for tuning ORR activity of a single Fe-$N_4$ site by controlling electron-withdrawing/donating properties of a carbon plane. *J. Am. Chem. Soc.* **141**, 6254-6262 (2019).
26. Meng, T., Hao, Y. N., Zheng, L. R. & Cao, M. H. Organophosphoric acid-derived CoP quantum dots@S,N-codoped graphite carbon as a trifunctional electrocatalyst for overall water splitting and Zn-air batteries. *Nanoscale* **10**, 14613-14626 (2018).
27. Wang, Y. et al. $Fe_3O_4$@N-doped interconnected hierarchical porous carbon and its 3D integrated electrode for oxygen reduction in acidic media. *Adv. Sci.* **7**, 2000407 (2020).
28. Han, A. J. et al. A polymer encapsulation strategy to synthesize porous nitrogen-doped carbon-nanosphere-supported metal isolated-single-atomic-site catalysts. *Adv. Mater.* **30**,1706508 (2018).
29. Li, F. et al. Boosting oxygen reduction catalysis with abundant copper single atom active sites. *Energ. Environ. Sci.* **11**, 2263-2269 (2018).
30. Chen, Y. J. et al. Enhanced oxygen reduction with single-atomic-site iron catalysts for a zinc-air battery and hydrogen-air fuel cell. *Nat. Commun.* **9**, 5422 (2018).
31. Pan, Y. et al. A bimetallic Zn/Fe polyphthalocyanine-derived single-Atom Fe-$N_4$ catalytic site: A superior trifunctional catalyst for overall water splitting and Zn-air batteries. *Angew. Chem. Int. Ed.* **57**, 8614-8618 (2018).
32. Chen, Y. J. et al. Isolated single iron atoms anchored on N-doped porous carbon as an efficient electrocatalyst for the oxygen reduction reaction. *Angew. Chem. Int. Ed.* **56**, 6937-6941 (2017).
33. Kim, S. J. et al. Defect-free encapsulation of $Fe_0$ in 2D fused organic networks as a durable oxygen reduction electrocatalyst. *J. Am. Chem. Soc.* **140**, 1737-1742 (2018).
34. Liu, Q. T., Liu, X. F., Zheng, L. R. & Shui, J. L. The solid-phase synthesis of an Fe-N-C electrocatalyst for high-power proton-exchange membrane fuel cells. *Angew. Chem. Int. Ed.* **57**, 1204-1208 (2018).
35. Zhang, Z. P., Sun, J. T., Wang, F. & Dai, L. M. Efficient oxygen reduction reaction (ORR) catalysts based on single iron atoms dispersed on a hierarchically structured porous carbon framework. *Angew. Chem. Int. Ed.* **57**, 9038-9043 (2018).
36. Li, Q. H. et al. Fe isolated single atoms on S, N codoped carbon by copolymer pyrolysis strategy for highly efficient oxygen reduction reaction. *Adv. Mater.* **30**, 1800588 (2018).
37. Lu, Z. et al. An isolated zinc-cobalt atomic pair for highly active and durable oxygen reduction. *Angew. Chem. Int. Ed.* **58**, 2622-2626 (2019).
38. Peng, P. et al. A pyrolysis-free path toward superiorly catalytic nitrogen-coordinated single atom. *Sci. Adv.* **5**, eaaw2322 (2019).
39. Chen, P. Z. et al. Atomically dispersed iron-nitrogen species as electrocatalysts for bifunctional oxygen evolution and reduction reactions. *Angew. Chem. Int. Ed.* **56**, 610-614 (2017).
40. Jiang, W. J. et al. Understanding the high activity of Fe-N-C electrocatalysts in oxygen reduction: Fe/$Fe_3C$ nanoparticles boost the activity of Fe-$N_x$. *J. Am. Chem. Soc.* **138**, 3570-3578 (2016).

41. Wang, Y. C. et al. S-doping of an Fe/N/C ORR catalyst for polymer electrolyte membrane fuel cells with high power density. *Angew. Chem. Int. Ed.* **54**, 9907-9910 (2015).
42. Yang, L. et al. Unveiling the high-activity origin of single-atom iron catalysts for oxygen reduction reaction. *P. Natl. Acad. Sci. USA* **115**, 6626-6631 (2018).
43. Shen, H. J. et al. Synergistic effects between atomically dispersed Fe-N-C and C-S-C for the oxygen reduction reaction in acidic media. *Angew. Chem. Int. Ed.* **56**, 13800-13804 (2017).
44. Zhang, H. G. et al. High-performance fuel cell cathodes exclusively containing atomically dispersed iron active sites. *Energ. Environ. Sci.* **12**, 2548 (2019).
45. Shui, J. L., Chen, C., Grabstanowicz, L., Zhao, D. & Liu, D. J. Highly efficient nonprecious metal catalyst prepared with metal-organic framework in a continuous carbon nanofibrous network. *P. Natl. Acad. Sci. USA* **112**, 10629-10634 (2015).
46. Wang, J. et al. Design of N-coordinated dual-metal sites: A stable and active Pt-free catalyst for acidic oxygen reduction reaction. *J. Am. Chem. Soc.* **139**, 17281-17284 (2017).
47. Zhang, H. G. et al. Single atomic iron catalysts for oxygen reduction in acidic media: Particle size control and thermal activation. *J. Am. Chem. Soc.* **139**, 14143-14149 (2017).
48. Han, Y. H. et al. Hollow N-doped carbon spheres with isolated cobalt single atomic sites: Superior electrocatalysts for oxygen reduction. *J. Am. Chem. Soc.* **139**, 17269-17272 (2017).
49. Yi, J. D. et al. Atomically dispersed iron-nitrogen active sites within porphyrinic triazine-based frameworks for oxygen reduction reaction in both alkaline and acidic media. *ACS Energy Lett.* **3**, 883-889 (2018).
50. Wan, G. et al. Engineering single-atom cobalt catalysts toward improved electrocatalysis. *Small* **14**, 1704319 (2018).
51. Li, J. Z. et al. Thermally driven structure and performance evolution of atomically dispersed $FeN_4$ sites for oxygen reduction. *Angew. Chem. Int. Ed.* **58**, 18971 (2019).
52. Gao, L. Q. et al. Hydrogen etching induced hierarchical meso/micro-pore structure with increased active density to boost ORR performance of Fe-N-C catalyst. *J. Energy. Chem.* **35**, 17-23 (2019).
53. Zeng, H. J., Wang, W., Li, J., Luo, J. & Chen, S.L. In situ generated dual-template method for Fe/N/S Co-doped hierarchically porous honeycomb carbon for high-performance oxygen reduction. *ACS Appl. Mater. Interfaces* **10**, 8721-8729 (2018).
54. Xiao, M. L. et al. Microporous framework induced synthesis of single-atom dispersed Fe-N-C acidic ORR catalyst and its in situ reduced Fe-$N_4$ active site identification revealed by X-ray absorption spectroscopy. *ACS Catal.* **8**, 2824-2832 (2018).
55. Fu, X. G. et al. In situ polymer graphenization ingrained with nanoporosity in a nitrogenous electrocatalyst boosting the performance of polymer-electrolyte-membrane fuel cells. *Adv. Mater.* **29**,1604456 (2017).
56. Ratso, S. et al. Synthesis of highly-active Fe-N-C catalysts for PEMFC with carbide-derived carbons. *J. Mater. Chem. A* **6**, 14663-14674 (2018).
57. Chung, H. T. et al. Direct atomic-level insight into the active sites of a high-performance PGM-free ORR catalyst. *Science* **357**, 479-483 (2017).
58. Sa, Y. J. et al. A general approach to preferential formation of active Fe-$N_x$ sites in Fe-N/C electrocatalysts for efficient oxygen reduction reaction. *J. Am. Chem. Soc.* **138**, 15046-15056 (2016).
59. Zitolo, A. et al. Identification of catalytic sites for oxygen reduction in iron- and nitrogen-doped graphene materials. *Nat. Mater.* **14**, 937 (2015).

60. Zion, N., Cullen, D. A., Zelenay, P. & Elbaz, L. Heat-treated aerogel as a catalyst for the oxygen reduction reaction. *Angew. Chem. Int. Ed.* **59**, 2483-2489 (2020).
61. Xu, H. X., Cheng, D. J., Cao, D. P. & Zeng, X. C. A universal principle for a rational design of single-atom electrocatalysts. *Nat. Catal.* **1**, 339-348 (2018).
62. Perdew, J. P., Burke, K. & Ernzerhof, M. Generalized gradient approximation made simple. *Phys. Rev. Lett.* **77**, 3865-3868 (1996).
63. Saputro, A. G., Fajrial, A. K., Agusta, M. K. & Dipojono, H. K. Density functional study on the formation of sulfur-doped configuration on the active site of pyrolyzed Fe/N/C catalyst. *J. Phys. Conf. Ser.* **1204**,012119 (2019).
64. Ni, B. X. et al. Optimized enhancement effect of sulfur in Fe-N-S codoped carbon nanosheets for efficient oxygen reduction reaction. *ACS Appl. Mater. Interface* **12**, 23995-24006 (2020).
65. Li, S., Cheng, C., Zhao, X. J., Schmidt, J. & Thomas, A. Active salt/silica-templated 2D mesoporous FeCo-$N_x$-carbon as bifunctional oxygen electrodes for zinc-air batteries. *Angew. Chem. Int. Ed.* **57**, 1856-1862 (2018).
66. Tang, C., Wang, B., Wang, H. F. & Zhang, Q. Defect engineering toward atomic Co-$N_x$-C in hierarchical graphene for rechargeable flexible solid Zn-air batteries. *Adv. Mater.* **29**, 1703185 (2017).
67. Shinde, S. S. et al. Unveiling dual-linkage 3D hexaiminobenzene metal-organic frameworks towards long-lasting advanced reversible Zn-air batteries. *Energ. Environ. Sci.* **12**, 727-738 (2019).
68. Zou, L. L., Hou, C. C., Liu, Z., Pang, H. & Xu, Q. Superlong single-crystal metal-organic framework nanotubes. *J. Am. Chem. Soc.* **140**, 15393-15401 (2018).
69. Wang, J. et al. Synergistic effect of well-defined dual sites boosting the oxygen reduction reaction. *Energ. Environ. Sci.* **11**, 3375-3379 (2018).
70. Han, X. P. et al. Atomically dispersed binary Co-Ni sites in nitrogen-doped hollow carbon nanocubes for reversible oxygen reduction and evolution. *Adv. Mater.* **31**, 1905622 (2019).
71. Yu, P. et al. Co Nanoislands rooted on Co-N-C nanosheets as efficient oxygen electrocatalyst for Zn-air batteries. *Adv. Mater.* **31**, 1901666 (2019).
72. Yang, J. et al. In-situ cobalt and nitrogen doped mesoporous graphitic carbon electrocatalyst via directly pyrolyzing hyperbranched cobalt phthalocyanine for hydrogen evolution reaction. *Electrochim. Acta* **262**, 48-56 (2018).
73. Yang, M. X. et al. Synchronously integration of Co, Fe dual-metal doping in Ru@C and CDs for boosted water splitting performances in alkaline media. *Appl. Catal. B-Environ.* **267**, 118657 (2020).
74. Liang, H. W. et al. Molecular metal-$N_x$ centres in porous carbon for electrocatalytic hydrogen evolution. *Nat. Commun.* **6**, 7992 (2015).
75. Cao, L.L. et al. Identification of single-atom active sites in carbon-based cobalt catalysts during electrocatalytic hydrogen evolution. *Nat. Catal.* **2**, 134-141 (2019).
76. Wan, J. W. et al. In situ phosphatizing of triphenylphosphine encapsulated within metal-organic frameworks to design atomic $Co_1$-$P_1N_3$ interfacial structure for promoting catalytic performance. *J. Am. Chem. Soc.* **142**, 8431-8439 (2020).
77. Pan, Y. et al. Core-shell ZIF-8@ZIF-67-derived CoP nanoparticle-embedded N-doped carbon nanotube hollow polyhedron for efficient overall water splitting. *J. Am. Chem. Soc.* **140**, 2610-2618 (2018).
78. Tiwari, J. N. et al. High-performance hydrogen evolution by Ru single atoms and nitrided-Ru nanoparticles implanted on N-doped graphitic sheet. *Adv. Energy Mater.* **9**, 1900931 (2019).

79. Creus, J. et al. Ruthenium nanoparticles supported on carbon microfibers for hydrogen evolution electrocatalysis. *Eur. J. Inorg. Chem.* **2019,** 2071-2077 (2019).
80. Li, J. C. et al. Synergistic effect of ultrafine nano-Ru decorated cobalt carbonate hydroxides nanowires for accelerated alkaline hydrogen evolution reaction. *Electrochim Acta* **331**, 135367 (2020).
81. Wu, X. J. et al. Metal-support interaction boosted electrocatalysis of ultrasmall iridium nanoparticles supported on nitrogen doped graphene for highly efficient water electrolysis in acidic and alkaline media. *Nano Energy* **62**, 117-126 (2019).
82. Li, F. et al. Balancing hydrogen adsorption/desorption by orbital modulation for efficient hydrogen evolution catalysis. *Nat. Commun.* **10**, 4060 (2019
83. Zhang, Z. R. et al. Electrochemical deposition as a universal route for fabricating single-atom catalysts. *Nat. Commun.* **11**, 1215 (2020).
84. Li, D. B. et al. Atomically dispersed platinum supported on curved carbon supports for efficient electrocatalytic hydrogen evolution. *Nat. Energy* **4**, 512-518 (2019).
85. Jiang, K. et al. Single platinum atoms embedded in nanoporous cobalt selenide as electrocatalyst for accelerating hydrogen evolution reaction. *Nat. Commun.* **10**, 1743 (2019).
86. Zhang, Z. Q. et al. The simplest construction of single-site catalysts by the synergism of micropore trapping and nitrogen anchoring. *Nat. Commun.* **10**, 1657 (2019).
87. Li, Z. et al. In situ formed $Pt_3Ti$ nanoparticles on a two-dimensional transition metal carbide (MXene) used as efficient catalysts for hydrogen evolution reactions. *Nano. Lett.* **19**, 5102-5108 (2019).
88. Bernsmeier, D. et al. Outstanding hydrogen evolution performance of supported Pt nanoparticles: Incorporation of preformed colloids into mesoporous carbon films. *J. Catal.* **369**, 181-189 (2019).
89. Shen, Y., Lua, A. C., Xi, J. Y. & Qiu, X. P. Ternary platinum-copper-nickel nanoparticles anchored to hierarchical carbon supports as free-standing hydrogen evolution electrodes. *ACS Appl. Mater. Interfaces* **8**, 3464-3472 (2016).
